# Competencies and skills of nursing undergraduate students as educators in child health care

**DOI:** 10.1590/1980-220X-REEUSP-2025-0385en

**Published:** 2026-06-01

**Authors:** Thays Mylena Lima da Silva, Débora Maria Santana da Silva, Gracielly Karine Tavares Souza, Cândida Maria Rodrigues dos Santos, Eliane Maria Ribeiro de Vasconcelos, Estela Maria Leite Meirelles Monteiro

**Affiliations:** 1 Universidade Federal de Pernambuco, Recife, PE, Brazil.

**Keywords:** Competency-Based Education, Social Skills, Nursing, Child Health

## Abstract

**Objective:**

To analyze the perception of undergraduate nursing students regarding the competencies and skills required for conducting health education in the context of child health.

**Method:**

This is an exploratory, descriptive, and qualitative study. It was developed in a virtual environment through interviews on *Google Meet*, between March and May 2024. The study population consisted of 30 Nursing students from two public universities in Pernambuco. The interviews had a maximum duration of 30 minutes, with six open-ended questions. The transcripts were made on the platform *Transkryptor*, unified into one document and analyzed using the software *NVivo*.

**Results:**

Based on the data analysis, four categories were developed: Understanding the competencies and skills required in health education; Specific requirements for working as a health educator with children; Strategies and resources for health education with children; and Contribution of a Nursing degree to health education initiatives.

**Conclusion:**

Nursing undergraduate students’ perception of the skills and abilities needed for health education in the context of childhood reveals a deficit in their curriculum, especially regarding preparation for working as health educators for children.

## INTRODUCTION

Health education, grounded in the principles of popular health education, emerges with a commitment to ensuring its application in the daily practice of healthcare, emphasizing its promotion, contextualization, and the intertwining of scientific and popular knowledge, considering social determinants and local cultures to effectively promote care, equity, and social participation^(1)^.

In this sense, over the years, health education has incorporated various teaching methodologies and technologies, with the aim of consolidating itself in the areas of health and Pedagogy. In healthcare, its main goal is to promote health and change lifestyle habits in different populations and healthcare contexts, as well as to provide training for critical and competent professionals regarding the practice of their profession and their stance on social ills, through education, ratifying health policies and the population’s needs^(2)^.

The National Curriculum Guidelines (*DCN*) establish that nursing education should be based on humanistic, scientific, and ethical-political principles, integrating teaching, research, and extension, and ensuring experiences that favor the development of technical-scientific and sociocultural competencies for comprehensive care^(3)^. From this perspective, popular health education is configured as a cross-cutting and methodological axis in training, as it stimulates problematizing educational praxis and social commitment to equity, citizenship, and the transformation of local realities. Thus, undergraduate studies, especially in health-related fields, become an important tool for developing skills and solidifying knowledge related to educational practices in health^(4)^.

According to the literature, competencies can be defined as the grouping of knowledge, skills, and attitudes required for the development of tasks and for acting in problem-solving, enabling the individual to perform a given profession^(5)^. Accordingly, skills can be defined as tools that enhance technical ability in a given professional field^(6)^.

However, regarding the development of competencies and skills for the care and promotion of the child population’s health, nurses or nursing students sometimes experience a feeling of insecurity, since the management and communication of health issues with this population must be individualized according to age group and stage of development^(7)^. Furthermore, the nurse educator in child health must develop communicative, playful, and dialogical skills that promote the empowerment of these children as active agents in learning about self-care and health promotion^(8)^. In this respect, for health education in the childhood age group to be effective, it is necessary to develop competencies and skills since undergraduate studies^(9)^.

Therefore, when considering health education for children, one should understand that the development of this target audience is dynamic and that they are not merely passive recipients of information, but individuals capable of developing autonomy through language and experimentation in the world around them^(7)^. Moreover, the findings indicate an insufficient definition, a limited national contextualization, and a curricular misalignment of the competencies and skills that nursing under-graduates need to act as health educators in child care—both due to the lack of recent Brazilian evidence in school health and due to international divergences and a mismatch between training and practice^(10,11,12)^. Therefore, this study aims to analyze the perception of undergraduate Nursing students regarding the competencies and skills required for conducting health education in the context of child health.

## METHOD

### Design of Study

This is a qualitative, exploratory, and descriptive study based on semi-structured interviews. Data were analyzed using thematic content analysis, as proposed by Bardin^(13)^. The study report followed the recommendations of Consolidated Criteria for Reporting Qualitative Research (COREQ)^(14)^, with the aim of ensuring transparency and completeness in the presentation of qualitative research. All applicable items were considered in accordance with the nature and objectives of the research.

### Local

The study was developed in a virtual environment, using the platform *Google Meet*, with a view to making interview times more flexible, given that most undergraduate Nursing courses are held full-time.

### Population

The study population consisted of undergraduate Nursing students from two public universities in the state of Pernambuco, Brazil, who shared their opinions on the topic discussed.

### Selection criteria

The study included undergraduate nursing students regularly enrolled in Higher Education Institutions (HEIs) who, at the time of data collection, had already completed the Child Health module or course.

### Data Collection

Data collection took place from March to May 2024. The selection of participants was carried out using the snowball sampling technique, which consists in using a reference individual, called a seed, who indicates a participant who meets the research participation criteria^(12)^. To identify the reference individual, an invitation letter was sent, containing information about the study’s objectives and participation. This letter was sent via email to a representative from each class that met the selection criteria, as provided by the secretariat of the participating HEIs. The representatives agreed to participate and nominated a new member; the remaining contacts were mediated through social media (*Instagram* and *WhatsApp*).

Following the initial contact with the participant, an individual interview was scheduled, taking into account the best day and time. At the time of the interview, the Free Informed Consent Form (FICF) and the Image and Testimony Authorization Form were read aloud to the participant. After formally agreeing to participate in the study, the student was instructed to complete a form via *Google Forms,* which contained 11 objective questions with sociodemographic data, to outline the participants’ profile. The researcher also used a script for observing subjective data, primarily gleaned from tone of voice and response time, which required sensitivity to analyze interest, articulation, mastery, and confidence.

During data collection in a virtual environment, frequent interruptions occurred only in one interview, due to Internet instability, and it was agreed that rescheduling was necessary, at which point participation became possible again. Therefore, there was no sample loss.

Subsequently, the interview began with screen recording, with the participant having the option to turn the webcam on*.* It should be noted that the interviews had a maximum duration of 30 minutes, during which six subjective questions were asked based on the research objectives, namely:

“*What do you understand by competencies in health education? How would you explain the difference between competencies and skills? In your opinion, what competencies are necessary to develop health education actions for children? Based on your experience, what strategies do you use (or did you use) to develop health education actions for children? Did you learn any of these during your undergraduate studies? If you were to conduct an educational activity for children, what materials/tools would you use? From your point of view, how does a degree in Nursing contribute to the development of these skills and abilities?”*

To test the clarity of the interview script, a pilot study was conducted with three participants, with the aim of observing the comprehensibility of the questions. Since no revision of the script was necessary, the participants from the pilot application were included in the sample. Data collection was interrupted when responses reached saturation during the interviews, as recommended by the methodological approach, in which, in qualitative research, it is more important to encompass the dimensions of the chosen topic than the number of participants^(15)^.

### Data Analysis and Treatment

The participants’ accounts, in audio format, were transcribed in full with the aid of the platform *Transcryptor.* However, it was necessary to review the material to ensure the preservation of the literal content of the dialogue. Following the organization of the collected data, the analysis began using the proposed Content Analysis method, which aims to categorize and interpret the study’s findings. According to Bardin^(13)^, this practice can occur in three phases: pre-analysis; exploration of the material; and treatment of the results. In the pre-analysis, the researcher combined the participants’ accounts with records of subjective data perceived during the interview, in which each question initially corresponded to a variable, and subsequently, the data were unified into a document. Subsequently, the material exploration phase was carried out with the assistance of the software *NVivo* ^(16)^, aiming to facilitate the identification of units that make up the description of the highlighted characteristics of the content, contributing to the classification and aggregation in the definition of categories^(15)^. Finally, an inductive analysis of the compiled and organized narratives was carried out using semantic similarity^(15)^.

### Ethical Aspects

All ethical aspects described in Resolution No. 466/2012 of the National Health Council (*CNS*) were followed, and the study was approved by the Research Ethics Committee (*CEP*) of the Faculdade Frassinetti do Recife (*FAFIRE*), through opinion No. 6,744,689 and CAAE No. 78472224.6.0000.5586. Data collection followed all recommendations of Circular Letter No. 2/2021 from the National Research Ethics Committee (*CONEP*) for research in a virtual environment, and all participants signed the FICF. To preserve the participants’ anonymity, their statements were identified with the letter “P” followed by the Arabic numeral.

## RESULTS

The study involved 30 undergraduate nursing students from the Universidade Federal de Pernambuco (*UFPE*) and the Universidade de Pernambuco (*UPE*). Thus, the participation of 13 undergraduate students (43%) from UPE and 17 undergraduate students (57%) from UFPE was observed. The students’ profile was identified through the sociodemographic form completed before the start of the interviews, as described in Table 1.

**Table 1:** Distribution of the sociodemographic profile of participants – Recife, PE, Brazil (2024).

Factor evaluated	n	%
Age range		
18 to 21 years	10	33
22 to 25 years	15	50
26 to 30 years	5	17
Sex		
Female	25	83
Male	5	17
Race		
White	13	43
Brown	12	40
Black	5	17
University		
Universidade Federal de Pernambuco (UFPE)	17	57
Universidade de Pernambuco (UPE)	13	43
Academic term		
6th period	2	7
7th period	2	7
8th period	5	17
9th period	16	53
10th period	4	13
Displaced, but has already taken a course/module on child health	1	3
Has a previous degree?		
Yes	1	3
No	29	97
Participates in extension projects, activities, and workshops that work with children?		
Yes	14	47
No	16	53
Has research projects (PIBIC, PET, others) focused on children?		
Yes	4	13
No	26	87

Source: Prepared by the authors (2024).

It was found that the majority were female (83%) with a mean age of 23 years. Regarding race, 44% self-identify as white, 40% as mixed-race, and 17% as black. Furthermore, it was found that the majority of participants (54%) were in their 9th semester at the time of the research, and among these, only 1 (3%) had a previous degree. Of the total participants, 14 (47%) participated in extension projects, activities and workshops aimed at children, and 13% participated in research projects aimed at this audience.

Based on the promptness and articulation of the responses, the researcher observed greater fluency and mastery of the subject matter on the part of the undergraduate students from one particular HEI compared to the other, as they reported greater practical experience in encouraging the production and application of playful educational materials about health with children. At the other institution, only those who had experience in extension projects, also involving the production and application of playful educational materials with this audience, demonstrated greater fluency, articulation, and mastery in their responses.

Based on data analysis and the participants’ statements, four categories were developed: Understanding the competencies and skills required in health education; Specific requirements for working as a health educator with children; Strategies and resources for health education with children; and Contribution of a Nursing degree to health education initiatives.

### Understanding the Competencies and Skills in Health Education

When expressing their empirical knowledge to distinguish the competencies and skills required of health professionals, some undergraduate students related competencies in health education to the theoretical knowledge acquired during their training, while in the definition of skill, an association with practical performance was observed, as demonstrated in the following discourse:

*“Competence is the knowledge I have, and skill is my ability to perform educational actions.” For example, each audience we care for requires a specific language and a particular way of behaving.* (...). *So, the way we approach the person being assisted is a skill we perform within the scope of health education.” (P13)*

During the recording of the reports, some graduating students were also identified who had some difficulty expressing their knowledge about the competencies and skills required for the educational role of nurses in healthcare, as demonstrated below:

*“I never thought about it, about the difference between competence and skill, but I believe that skill is something inherent in you, it’s the ability to communicate, to be understood in a given situation.” “Competence, in turn, should be related to what you have studied; that is, it’s having the ability to pass on what you’ve learned, to pass on your knowledge.” (P5)*

*“For me, these terms are very intertwined, and I can’t distinguish between what would be a competence and what would be a skill in this matter of education.” (P3)*

### Specific Skills Required to Work as a Health Educator with Children

To work as health educators with children, the undergraduates brought a perspective related to the planning of educational activities, which should have as their main goal the use of playful language adapted to the child audience, mastery of scientific knowledge on the subject to be addressed, and observation of the child’s profile and environment.

*“I believe that creativity and playful, adapted, and accessible language are key, because we are used to using many technical terms; due to force of habit, we often don’t ‘police’ ourselves to use more accessible language”* (...). *If we use technical terms, the audience won’t be able to understand the information being conveyed, and it won’t have the desired impact if it were adapted for a specific audience.” (P2)*

*“First, it’s about understanding the specific characteristics of the audience we’re dealing with, understanding how children are situated within that context, what they like, what interests them.” “Because, within the children’s audience, we have a variety of interests, since each age group has its own developmental milestones.” (P27)*

*“It requires sensitivity, because you can read the children’s body language and notice if they are building a line of reasoning along with you.”* (...) *I believe that only in this way will health education have a positive effect, because, by understanding the importance of the subject, the child will encourage parents, guardians, friends, etc.” (P25)*

Some students brought a perspective to the application of health education actions also in the hospital context, seeking not only to involve the child, but also to include the caregiver, providing adaptive scenarios for clarifications and guidance, in a pleasant and creative way, driven by a feeling of empathy.

*“*(...)*Having the ability to identify the target audience for your campaign. Speaking specifically about children, I believe it’s more complex because this process needs to be done in a playful way so that they can understand it. Children already have a cultural fear of hospitals, nurses, doctors, etc., so I think it’s difficult to break that mindset.” (P30)*

### Strategies and Resources for Health Education with Children

Regarding the use of strategies and resources to enable the teaching-learning process in the context of children, participants pointed out that knowledge of the child’s daily reality is essential to promote the exchange of knowledge with the target audience through playful strategies, the use of active methodologies, and didactic resources, which, according to the research participants, guarantee active participation.

*“So, I think developing an active methodology is the key: that the child participates, that it’s playful, that it’s dynamic, bringing in some everyday things so that the child can identify with that situation, that it’s more within their reality.” At times, I used a circle time format where the children could speak, give their opinions on the situation, and provide examples, even before we started talking about the subject. Including them in this dialogue is important so that they become more interested in the subject.” (P1)*

*“Games, to get the child actively involved in the activity.”* (...) *To do more than just explain a subject; for example, to use games, theatrical performances, strategies that place the child as the protagonist of that context.” (P16)*

It was observed that there is a preference for the use of educational games as a teaching resource today. According to the participants, the use of these resources helps to connect the content being taught with the daily lives of the audience, and also moves away from the traditional teaching-learning process, bringing play as a form of learning.

*“I remember the child health internship, because when we were in the ward, we thought about working on the topic of personal hygiene with the hospitalized children.” So, we used a game strategy, like a memory game, and some pictures for them to color, because while the children were playing, they were learning about the topic.” (P21)*

*“Games, competitions and figures.” Children love games, especially those that involve rewards, and games and competitions encourage them to participate spontaneously.” (P29)*

Furthermore, it was observed, as a reflection of the COVID-19 pandemic, that some undergraduate students adapted their playful strategies, previously used in person, to the online context, as a way to continue disseminating health information to children.

*“I only had the opportunity to carry out an educational activity for children once; however, since it was during the pandemic, the activity had to take place online.” So, the strategy we used was colorful and playful slides, and we asked them to make a PET bottle puppet beforehand to simulate CPR compressions. Despite being online, I believe that using this strategy was quite beneficial, as they interacted a lot, especially at the end when we did the true or false question activity. Unfortunately, I’ve never had the opportunity to conduct health education with children in person, but I believe that implementing these strategies in person would capture the attention of this audience more effectively.” (P26)*

### Contribution of the Nursing Undergraduate Course to Health Education Initiatives

In respect of the contribution of a Nursing degree to the development of competencies and skills geared towards health education actions, the graduating students highlighted the importance of being involved in extracurricular activities that stimulate hands-on experience, especially those aimed at children, as they contribute to safety in the development of educational activities. It is observed that, according to the participants’ view, the acquisition of competencies and skills to deal with health education for children is rarely addressed in the subjects of the curriculum.

*“In my experience during my undergraduate studies, I didn’t see anything that addressed health education for children, the use of play, and the strategies we can use with this audience, but rather in extracurricular activities, such as extension projects.” (P14)*

*“The Nursing degree helps me get closer to projects focused on this theme because, in the curriculum, at least from my point of view, it deals a lot with children’s diseases and conditions, being very focused on the care aspect.” And when we have the help of extension projects or academic leagues, we are able to develop these skills better. Furthermore, the tutoring provided by the students themselves allows us to understand what it’s like to plan and execute a lesson, as well as helping us in the process of passing on the content we’ve learned.” (P27)*

Furthermore, the graduates also raised the need for adjustments to the curriculum aimed at improving the development of these competencies and skills. Among the points listed for improvement, the following stand out: reducing the focus on hospital care practices; including classes aimed at active strategies and methodologies that can be used in health education; discussions about popular education with the inclusion of children; as well as encouraging the development of educational activities with children, as described in the statements below:

*“Undergraduate studies focus heavily on the clinical aspect and forget that health education ranges from basic care to highly complex procedures.” And despite its importance, during my undergraduate studies I don’t recall having classes on health education strategies; so what I learned was through practice, through trial and error, and I refined it for different contexts.” (P22)*

## DISCUSSION

By stimulating critical thinking about competencies and skills in health education, the undergraduate students were able to present their understandings regarding the topic. With regard to competencies, there is an intrinsic relationship with the acquisition of scientific knowledge and the ability to transform it into content accessible to different audiences, which requires not only competencies but also educational skills. Thus, it can be stated that competencies encompass a set of knowledge, attitudes, motivations, self-perceptions, and skills that make educational action possible^(17)^.

Despite understanding the concept of health education, some students had difficulty correlating this understanding with the skills required of nursing professionals in the field of health promotion. Following a concept aligned with national literature and the *DCN*s, competence, within the scope of training, is understood as the mobilization of knowledge, skills, attitudes, and values that enable graduates to adopt a posture of leadership, communication, decision-making, and acting as a facilitator in educational actions^(3)^.

Although the view of the nurse as a health educator is ratified, there is not always an understanding of the competencies that this professional must develop for this role to be consolidated as a work tool. Based on findings from the literature, competencies should be related to clinical judgment, communication, person-centered care, equity, inclusion, ethics, evidence-based practice, health policies, and the social determinants of health^(18)^.

Regarding educational skills, the understanding extended to practical abilities, action, and the educational moment itself, being described as the capacity to transform scientific knowledge into popular knowledge through different methodologies. When reflecting on the skills required of nursing professionals in the performance of educational actions, it is observed that they refer to practical, cognitive, and socio-emotional dimensions that guide the mobilization of competencies in healthcare work. By encouraging the development of such skills during undergraduate studies, future professionals are enabled to perform better in both clinical and educational settings^(19,20)^.

Furthermore, it was noted that communication skills were the most frequently cited among the study participants. This understanding is reinforced by the literature, in which Daza-Betancourt et al.^(21)^ highlighted that communication skills play a central role, as they are essential, among other skills to be developed by nurses, in creating opportunities for building bonds, trust, and empathy, as well as facilitating the construction of an environment conducive to the exchange of information.

Regarding the category “Specific requirements for the performance of nursing professionals as health educators with children,” it was possible to note the students’ concern regarding the promotion of children’s health through playful, accessible educational activities adapted to the audience and the reality in which the children are inserted. When considering health education, the school environment stands out for enabling intersectoral action between health and education. Among healthcare professionals, nurses play a leading role as mobilizers and coordinators of educational initiatives, utilizing their competencies to promote prevention and protection against health problems, aiming to mitigate vulnerabilities and contribute to the holistic development of children and adolescents^(22)^.

By considering the social, economic, and health context in which the target audience is embedded, it is possible to plan an action that simulates daily life within that population, thus acting as a stimulating factor for the child to become a multiplier of health knowledge in the community. Although play acts as a facilitator in the teaching-learning process, it is necessary to go beyond accessible language and avoid technical terms; the child’s life context, opinions, interests, and reasoning must be considered to sensitize and motivate them by relating their experiences to the content being addressed^(23)^.

When planning health education activities, the facilitator, in addition to scientific knowledge about the topic to be addressed, must use methodologies that serve as bridges for knowledge building with the target audience, as well as anticipate and manage possible unforeseen events during the educational activity.

In line with the research findings, it is understood that, to carry out educational activities in a children’s setting, it is necessary to plan the educational moment considering the location, target audience, duration of the activity, and content, with the aim of promoting knowledge through the establishment of a relationship of trust^(24)^. Furthermore, when defining the target audience for educational activities as children, the health educator must consider the different approaches corresponding to the distinct stages of child development. This is because the failure of some educational actions is related to the generalization of materials and methods for different age groups, contrary to the literature, which advocates that the implementation of educational activities in childhood should consider the child’s developmental stages^(25)^.

For the development of the teaching-learning process in the context of children, the graduates highlighted the essential importance of linking educational action with the child’s daily life, using strategies such as puppet theater, theatrical performances, storytelling, parodies, educational videos, drawings, toys, music therapy, among others, since such playful strategies guarantee active participation and enable the exchange of knowledge with the target audience. From this perspective, the literature indicates that these strategies have learning potential, mainly because play allows for the simulation of scenarios and contexts where children can explore, ask questions, and build a repertoire on the subject matter^(26)^.

Among the educational strategies mentioned, in addition to those that simulate the child’s daily life, strategies that encourage children’s protagonism stood out, such as the creation of conversation circles, in which children are encouraged to speak, express opinions, and present examples. By using strategies that promote children’s protagonism, the success of the educational activity becomes evident, because, although the use of play materials is widespread, the success of the health intervention lies in the relationships established during the process, and is not limited to the use of objects during the educational moment^(23)^.

Moreover, among the resources used to ensure the active participation of children, the undergraduate students highlighted the use of educational games, both traditional and digital, with an emphasis on the development of virtual educational technologies. A preference was observed for the development and application of digital educational games with children, since they stimulate learning by valuing prior experiences, promote dialogue between facilitators and the target audience, and contribute to the development of emotional, cognitive, and motor skills, in addition to distancing themselves from traditional teaching methodologies^(27)^.

The research results also highlighted the impact of the COVID-19 pandemic on health promotion activities with children, making it necessary to reformulate the methodologies traditionally used to ensure the continuity of educational actions. In this context, the use of Information and Communication Technologies (ICTs) was introduced, which include electronic resources that allow communication, interaction and learning, such as educational software, educational videos, games, sites, images, enabling the promotion of health beyond physical barriers^(28)^.

The research findings revealed a limitation in the understanding of competencies and skills, as well as the strategies and resources to be used in educational activities with children, among undergraduate students who did not have extension experience or practical work. Integration into the school environment, listening to students’ needs, and fostering collaboration with professors and administrators contribute to greater safety and development of expertise in conducting educational activities, highlighting the importance of the university’s threepronged approach.

The integration of teaching, research, and extension promoted within the academic environment was emphasized as a key contribution to the development of skills and abilities geared towards health education actions, since it allows undergraduate students to engage in theoretical and practical scenarios, fostering a multidimensional vision, developing a critical clinical perspective, and contextualizing knowledge for social transformation^(29)^. Additionally, the undergraduate students mentioned the importance of academic tutoring as a strategy to enhance the knowledge acquired in the subject and develop teaching-learning skills, since the purpose of tutoring is to clarify doubts regarding the area of knowledge, assist in indepth theoretical studies, and reinforce practical skills learned during the course^(30)^.

The students’ dissatisfaction with the curriculum is noteworthy, underscoring the greater focus of the subjects on diseases and conditions and the distancing from the perspective of popular health education, especially as it relates to children. The speeches evidenced the need for curricular adjustments, considering that, to better train future nurses, the undergraduate curriculum must be aligned with the transformations in the provision of health care for different populations. In this sense, the academic environment is configured as a space for transformation and alignment with current and future needs in the field of health^(17)^.

Although the development of nursing competencies and skills occurs gradually, based on the accumulation of experiences with different populations and the reinterpretation of knowledge in the face of new situations, students reported difficulty and insecurity in promoting educational activities for children, due to the greater focus of the undergraduate program on adults and older people. In this sense, to reduce such insecurity, the students should be encouraged during their training to develop autonomy in acquiring health knowledge, to improve communication, to strengthen interpersonal relationships, and to be problem-solving capable^(31)^.

For this to occur, HEIs can adopt active learning methodologies in professional training, since they simulate or immerse the student in the field of work with the aim of providing opportunities to acquire skills in health^(32)^. Among these strategies, the use of digital educational technologies stands out, as they promote the cognitive and attitudinal development necessary for the context of health promotion, besides diversifying the teaching-learning process^(30)^.

Although this study contributed to the understanding of the competencies and skills needed by nursing graduates to work as health educators in child health care, it was observed that the inclusion of only public universities and from a single state represents a limitation in terms of the depth of the subject matter. Likewise, gaps were identified in the literature regarding the conceptualization of competencies in health education and educational skills in the context of Nursing, which restricted a more specific and comparative discussion of the findings. Therefore, it is suggested that future studies broaden the understanding of these concepts, encompassing a greater number of HEIs, including private institutions, which concentrate a significant portion of Nursing students.

## CONCLUSION

Nursing undergraduates’ perception of the competencies and skills needed for health education in the context of childhood reveals a deficit in their curriculum, especially regarding preparation for working as health educators for children. Although they demonstrate an understanding of the importance of combining scientific content with playful methodologies to transform it into accessible and meaningful knowledge, the students revealed insecurity and difficulty in applying these practices, which is attributed to their limited experience working with children during their undergraduate studies.

However, for undergraduate students with theoretical and practical experience in a school context, competence can be defined as the integrated articulation between knowledge, skills, and attitudes, required for the professional to mobilize what they know (knowledge) and know how to do it (skill), in a contextualized manner and appropriate to the audience and the situation experienced. In addition, the importance of the student’s sensitivity in recognizing and valuing the specificities of each audience is emphasized, considering their real needs and the commitment to providing a playful and creative arena capable of motivating and stimulating the children’s participation.

In this context, extension activities, research groups, and academic tutoring stand out as privileged spaces for the development of these competencies, since they promote the insertion of students into real-world contexts and encourage the integration between theory and practice. Thus, such experiences fill curricular gaps and contribute to the training of nurses who are better prepared to act as health educators in child care.

## Data Availability

The entire dataset supporting the results of this study is available upon request to the corresponding author.

## References

[1] World Health Organization. (2024). Health literacy: fact sheet.. Geneva: WHO.

[2] Teixeira CP, Gasque KC, Guilam MC, Machado MF, Azevedo NG, Castro RF (2023). Educação na Saúde: fundamentos e perspectivas. Porto Alegre (RS): Editora Rede Unida;. Série Vivências em Educação na Saúde;.

[3] Brasil. (2024). Ministério da Educação. Conselho Nacional de Educação. Câmara de Educação Superior. Parecer CNE/CES n.º 443, de 3 de julho de 2024. Revisão das Diretrizes Curriculares Nacionais para os Cursos de Graduação em Enfermagem, licenciatura e bacharelado.. https://portal.mec.gov.br/docman/julho-2024/264151-pces443-24/filescielo.br.

[4] Dantas MCS, Silva MSL, Santos NCCB, Figueirêdo DSTO, Andrade LDF (2023). Educação em Saúde na formação acadêmica em enfermagem.. Espac Saude.

[5] Lopes OCA, Henriques SH, Soares MI, Celestino LC, Leal LA (2020). Competências dos enfermeiros na estratégia Saúde da Família.. Esc Anna Nery..

[6] Nogueira CCC, Soares AB, Monteiro M, Medeiros HCP (2020). Habilidades sociais e expectativas acadêmicas em estudantes de enfermagem.. Estud Pesqui Psicol..

[7] Rodrigues AMM, Cordeiro EGR, Moreira KNP, Pereira NS, Cruz TRF, Silva JE (2022). Desenvolvimento da leitura na educação infantil: o papel da ludicidade.. Pesqui Soc Desenvolv..

[8] Barbosa DCM, Nogueira MF, Figueiredo RCB (2022). O papel do enfermeiro na educação em saúde com crianças: desafios e possibilidades.. Rev Bras Enferm..

[9] Gómez-Cantarino S, Mazoteras-Pardo V, Rodríguez-Montejano J, Gradellini C, Cunha-Oliveira A, Ugarte-Gurrutxaga MI (2022). Theorising about child maltreatment: narrative review on health education models, conceptual frameworks and the importance of the information and communication technologies.. Front Psychol.

[10] Purabdollah M, Zamanzadeh V, Ghahramanian A, Valizadeh L, Mousavi S, Ghasempour M (2023). Competencies expected of undergraduate nursing students: a scoping review.. Nurs Open..

[11] Dantas HLL, Lima ABA, Mendes RCM, Linhares FMP, Sette GCS, Vasconcelos EMR (2025). Competences and skills of nurses in school health: a scoping review.. Rev Gaúcha Enferm..

[12] Tahan HM, Warren JI, Godfrey N, Zipp JS, McDonald RD (2025). New graduate nurse competencies—Part I: perceptions of academic faculty and practice leaders.. Nurs Outlook.

[13] Bardin L (2016). Análise de conteúdo..

[14] Souza VR, Marziale MH, Silva GT, Nascimento PL (2021). Tradução e validação para a língua portuguesa e avaliação do guia COREQ.. Acta Paul Enferm.

[15] Campos CJG, Saidel MGB (2022). Amostragem em investigações qualitativas: conceitos e aplicações ao campo da saúde.. Rev Pesqui Qualitativa..

[16] Tonin L, Lacerda MR, Brandão MAG, Nascimento JD, Souza JF, Rosso H (2023). Use of NVivo 10® software in concept analysis study.. Texto Contexto Enferm.

[17] Giddens J, Conroy S (2022). The Revised AACN *Essentials*: implications for nursing regulation.. J Nurs Regul..

[18] Lewis LS, Rebeschi LM, Hunt E (2022). Nursing education practice update 2022: competency-based education in nursing.. SAGE Open Nurs.

[19] Jacob MN, Rodrigues MM, Silva MCTM, Melaragno ALP, Fonseca AS, Assoni MAS, Mandelbaum MHS (2023). organizadores. Educação Permanente em Saúde..

[20] Leite DHB, Souza JFR, Silva MR, Jesus ASM, Lucena GF, Reis CC (2024). Habilidades e competências dos enfermeiros para atuarem em emergências pediátricas: estudo bibliométrico.. Revista JRG..

[21] Daza-Betancourt M, Ebratt-Rincón AL, Contreras-Quintero CA, Dueñas-Carrillo L, del Socorro Moreno Luna I, Palencia-Sánchez F (2024). Revisión rápida de la literatura sobre métodos de entrenamiento para adquirir habilidades blandas esenciales o sociales para el curso de la vida en jóvenes universitarios.. SocArXiv.

[22] Tavares EGFA, de Sousa PSA (2025). Educação em saúde nas escolas: a contribuição do enfermeiro no programa saúde na escola.. ARE..

[23] Ranyere J, Matias NCF (2023). A relação com o saber nas atividades lúdicas escolares.. Psicol Cienc Prof..

[24] González-Jaramillo V, Greca I, González S (2020). Revisión de las estrategias empleadas en el entorno escolar en la enseñanza de la nutrición humana.. Rev Chil Nutr.

[25] Garcia Lopes Merino MF, Machado Cruz Shibukawa B, Patricio Rissi G, Sousa da Fonseca B, Demitto Furtado M, Harumi Higarashi I (2022). Crianças e adolescentes com diabetes: ações educativas no desenvolvimento de habilidades para o autocuidado.. Nursing (São Paulo)..

[26] Soares AKF, Sá CHC, Lima RS, Barros MS, Coriolano MWL (2022). Comunicação em saúde nas vivências de discentes e docentes de Enfermagem: contribuições para o letramento em saúde.. Ciênc Saúde Coletiva..

[27] Godtsfriedt J, Godtsfriedt CES, Cardoso FL (2022). Efeito de uma intervenção com jogos digitais e webgames na motivação intrínseca em escolares.. J Phys Educ (Maringá)..

[28] Pavinati G, Lima LV, Soares JPR, Nogueira IS, Jaques AE, Baldissera VDA (2022). Tecnologias educacionais para o desenvolvimento de educação na saúde: uma revisão integrativa.. Arq Ciênc Saúde Unipar..

[29] Araujo de Albuquerque W, Moreno Amor AL (2025). Integração ensino, pesquisa e extensão na formação profissional em saúde: integration teaching, research and extension in professional health training.. RIES..

[30] Monteiro PVA, Costa MLP, Menezes RSP, Monte GLA, Lima GC (2021). Tecnologias educacionais na monitoria acadêmica de fisiologia humana e biofísica na graduação de enfermagem.. Rev UFPE on-line..

[31] Silva F, Sousa SS, Oliveira TS, Bezerra KS, Oliveira KM, Silva S (2021). Relato de estudantes universitários de enfermagem sobre a formação de competências em Saúde Coletiva.. Saúde Redes..

[32] Rodrigues PS, Marin MJS, Souza AP, Vernasque JRS, Grandin GM, Almeida KRV (2024). Perspectivas de estudantes e egressos sobre a aprendizagem baseada em problemas na formação de enfermeiros.. Cienc Saude Colet..

