# Supplementary material for: Competencies and skills of nursing undergraduate students as educators in child health care
Source: Rev Esc Enferm USP. 2026 Jun 1;60:e20250385. doi: 10.1590/1980-220X-REEUSP-2025-0385en (PMC13225613; doi:10.1590/1980-220X-REEUSP-2025-0385en)
Supplement: Translation

# Translation: Competências e habilidades de graduandos de enfermagem como educadores na atenção à saúde da criança

**DOI:** 10.1590/1980-220X-REEUSP-2025-0385pt

**Authors:** Thays Mylena Lima da Silva, Débora Maria Santana da Silva, Gracielly Karine Tavares Souza, Cândida Maria Rodrigues dos Santos, Eliane Maria Ribeiro de Vasconcelos, Estela Maria Leite Meirelles Monteiro

**Affiliations:** 1 Universidade Federal de Pernambuco, Recife, PE, Brasil.

**Keywords:** Educação Baseada em Competências, Habilidades Sociais, Enfermagem, Saúde da Criança

## Abstract

**Objetivo:**

Analisar a percepção dos graduandos de Enfermagem sobre as competências e habilidades necessárias para a realização da educação em saúde no contexto da saúde da criança.

**Método:**

O estudo caracteriza-se como exploratório, descritivo e qualitativo. Foi desenvolvido em ambiente virtual por meio de entrevistas no *Google Meet,* no período de março a maio de 2024. A população foi composta por 30 estudantes de Enfermagem de duas universidades públicas de Pernambuco. As entrevistas tiveram duração máxima de 30 minutos, com seis perguntas subjetivas. As transcrições foram realizadas na plataforma *Transkriptor,* unificadas em um documento e analisadas pelo *software NVivo*.

**Resultados:**

A partir da análise dos dados, foram elaboradas quatro categorias: Compreensão sobre as competências e habilidades em educação em saúde; Especificidades requeridas para atuar como educador em saúde com o público infantil; Estratégias e recursos para a educação em saúde com crianças; e Contribuição da graduação em Enfermagem para as ações de educação em saúde.

**Conclusão:**

A percepção dos graduandos de Enfermagem sobre as competências e as habilidades necessárias para a educação em saúde no contexto infantil evidencia a existência de um déficit na formação curricular, especialmente no que se refere à preparação para atuar como educadores em saúde de crianças.

## INTRODUÇÃO

A educação em saúde, embasada nos princípios da educação popular em saúde, emerge com o compromisso de assegurar sua aplicação no cotidiano da assistência à saúde, com ênfase em sua promoção, na contextualização e no entrelaçamento entre saberes científicos e populares, considerando os determinantes sociais e as culturas locais para, efetivamente, promover o cuidado, a equidade e a participação social^(1)^.

Nesse sentido, ao longo dos anos, a educação em saúde agregou diversas metodologias e tecnologias de ensino, com o intuito de se consolidar na área da saúde e na Pedagogia. Na saúde, seu intuito principal é a promoção da saúde e a mudança de hábitos de vida nos diferentes públicos e contextos de saúde, bem como proporcionar a formação de profissionais críticos e competentes no que se refere ao exercício da profissão e ao posicionamento frente às mazelas sociais, por meio da educação, ratificando políticas de saúde e as necessidades da população^(2)^.

As Diretrizes Curriculares Nacionais (DCN) estabelecem que a formação do enfermeiro deve ser pautada em princípios humanistas, científicos e ético-políticos, integrando ensino, pesquisa e extensão, e garantindo experiências que favoreçam o desenvolvimento de competências técnico-científicas e socio-culturais para o cuidado integral^(3)^. Nessa perspectiva, a educação popular em saúde configura-se como eixo transversal e metodológico na formação, pois estimula a práxis educativa problematizadora e o compromisso social com a equidade, a cidadania e a transformação das realidades locais. Desse modo, a graduação, em especial os cursos da área da saúde, passa a ser uma importante ferramenta para o desenvolvimento de habilidades e a solidificação de conhecimentos relacionados às práticas educativas em saúde^(4)^.

De acordo com a literatura, as competências podem ser definidas como o agrupamento de conhecimentos, habilidades e atitudes necessários para o desenvolvimento de tarefas e para a atuação na resolução de problemas, tornando o indivíduo capacitado para exercer determinada profissão^(5)^. Em consonância, as habilidades podem ser definidas como ferramentas que potencializam a capacidade técnica em determinada área de atuação profissional^(6)^.

Entretanto, no que se refere ao desenvolvimento de competências e habilidades para o cuidado e a promoção da saúde da população infantil, o enfermeiro ou o estudante de Enfermagem, por vezes, vivencia um sentimento de insegurança, pois o manejo e a comunicação em saúde realizados com esse público devem ser individualizados de acordo com a faixa etária e o estágio de desenvolvimento^(7)^. Além disso, o enfermeiro educador na saúde da criança deve desenvolver habilidades comunicativas, lúdicas e dialógicas que favoreçam o protagonismo dessas crianças como agentes ativas de aprendizagem sobre autocuidado e promoção da saúde^(8)^. Nesse sentido, para que a educação em saúde na faixa etária infantil seja efetiva, é necessário desenvolver competências e habilidades desde a graduação^(9)^.

Assim, ao pensar na educação em saúde para a população infantil, é necessário entender que o desenvolvimento desse público-alvo é dinâmico e que este não se configura como mero receptor de informações, mas como indivíduo capaz de desenvolver autonomia por meio da linguagem e da experimentação no mundo à sua volta^(7)^. Adicionalmente, os achados indicam uma insuficiente definição, uma contextualização nacional limitada e um desalinhamento curricular das competências e habilidades que os graduandos de Enfermagem precisam para atuar como educadores em saúde na atenção à criança — tanto pela ausência de evidências brasileiras recentes em saúde escolar quanto por divergências internacionais e por um descompasso entre formação e prática^(10,11,12)^. Em consonância, este estudo apresenta como objetivo analisar a percepção dos graduandos de Enfermagem sobre as competências e as habilidades necessárias para a educação em saúde no contexto da saúde da criança.

## MÉTODO

### Desenho do Estudo

Trata-se de um estudo qualitativo, exploratório e descritivo, baseado em entrevistas semiestruturadas. Os dados foram analisados por meio da análise de conteúdo temática, conforme a proposta de Bardin^(13)^. O relato do estudo seguiu as recomendações dos *Consolidated Criteria for Reporting Qualitative Research* (COREQ)^(14)^, com o objetivo de garantir a transparência e completude na apresentação da pesquisa qualitativa. Todos os itens aplicáveis foram contemplados conforme a natureza e os objetivos da pesquisa.

### Local

O estudo foi desenvolvido em ambiente virtual, por meio da plataforma *Google Meet*, com vistas a flexibilizar os horários das entrevistas, devido à maioria das aulas dos cursos de graduação de Enfermagem ocorrer em horário integral.

### População

A população foi composta por estudantes da graduação do curso de Enfermagem de duas universidades públicas do estado de Pernambuco – Brasil, que relataram suas opiniões sobre a temática abordada.

### Critérios de Seleção

Foram incluídos no estudo estudantes da graduação de Enfermagem regularmente matriculados em Instituições de Ensino Superior (IES) que, no momento da coleta, já tivessem cursado o módulo ou a disciplina de Saúde da Criança.

### Coleta de Dados

A coleta de dados ocorreu de março a maio de 2024. A seleção dos participantes ocorreu por meio da técnica de amostragem *snowball*, que consiste em utilizar um indivíduo de referência, denominado semente, o qual indica um participante que atenda aos critérios de participação da pesquisa^(12)^. Para captar o indivíduo de referência, foi enviada uma carta convite, que continha informações sobre os objetivos do estudo e sobre a participação. Essa carta foi enviada por meio de e-mail endereçado a um representante de cada turma que atendia aos critérios de seleção, mediante disponibilização pela secretaria das IES participantes. Os representantes aceitaram participar e indicaram um novo integrante; os demais contatos foram mediados pelas redes sociais (*Instagram* e *Whatsapp*).

A partir do contato inicial com o participante, era agendada a entrevista, de forma individual, considerando o melhor dia e horário. No momento da entrevista, foi lido, junto ao participante, o Termo de Consentimento Livre e Esclarecido (TCLE) e o Termo de Autorização de Imagem e Depoimento. Após o aceite formal para participar do estudo, o discente era instruído a responder a um formulário, por meio do *Google Forms*, que continha 11 perguntas objetivas com dados sociodemográficos, com o intuito de traçar o perfil dos participantes. A pesquisadora também utilizou um roteiro de observação de dados subjetivos, apreendidos principalmente pelo tom de voz e pelo tempo de resposta, requerendo sensibilidade para analisar o interesse, a articulação, o domínio e a segurança.

Durante a coleta de dados em ambiente virtual, ocorreu, em apenas uma entrevista, uma interrupção frequente por fragilidade na *Internet*, sendo acordada a necessidade de reagendamento, quando foi possível a nova participação. Desse modo não houve perda amostral.

Em seguida, iniciava-se a entrevista com a gravação da tela, sendo opcional ao participante a abertura da *webcam.* Destaca-se que as entrevistas tiveram duração máxima de 30 minutos, nas quais foram realizadas seis perguntas subjetivas baseadas nos objetivos da pesquisa, sendo elas:

*“O que você entende por competências em educação em saúde?”; “Como você explicaria a diferença entre competências e habilidades?”; “Na sua opinião, quais são as competências necessárias para desenvolver ações de educação em saúde para o público infantil?”; “Com base na sua experiência, quais estratégias você utiliza(ou) para desenvolver ações de educação em saúde para crianças? Alguma dessas você aprendeu durante a graduação?”; “Se você fosse realizar uma ação educativa para crianças, quais materiais/ferramentas você utilizaria?”; “No seu ponto de vista, como a graduação em Enfermagem contribui para o desenvolvimento dessas competências e habilidades?”*.

Para testar a clareza do roteiro de entrevista foi elaborado um estudo piloto com três participantes, com o intuito de observar a compreensibilidade das perguntas. Como não foi necessária a revisão do roteiro, os participantes da aplicação piloto foram incluídos na amostra. A coleta de dados foi interrompida quando foi constatada a saturação de respostas durante as entrevistas, conforme preconiza a abordagem metodológica, na qual, em pesquisas qualitativas, é mais importante abranger as dimensões do tema escolhido do que o número de participantes^(15)^.

### Análise e Tratamento dos Dados

Os relatos dos participantes, em forma de áudio, foram transcritos na íntegra com o auxílio da plataforma *Transkriptor.* No entanto, foi necessário revisar o material para assegurar a preservação do conteúdo literal das falas. Após a organização dos dados coletados iniciou-se a análise por meio da proposta de Análise de Conteúdo, a qual tem o objetivo de categorizar e interpretar os achados do estudo. Segundo Bardin^(13)^, essa prática pode ocorrer em três fases: a pré-análise; exploração do material; e tratamento dos resultados. Na pré-análise, a pesquisadora agregou os relatos dos participantes aos registros de dados subjetivos percebidos durante a entrevista, em que cada pergunta correspondeu, no primeiro momento, a uma variável, e posteriormente, os dados foram unificados em um documento. Na sequência, a etapa de exploração do material foi realizada com auxílio do *software NVivo* ^(16)^, visando facilitar a identificação de unidades que compõem a descrição das características evidenciadas do conteúdo, concorrendo para a classificação e a agregação na definição das categorias^(15)^. Por fim, foi realizada análise indutiva das narrativas compiladas e organizadas por meio da semelhança semântica^(15)^.

### Aspectos Éticos

Foram respeitados todos os aspectos éticos descritos na Resolução nº 466/2012 do Conselho Nacional de Saúde (CNS) e com aprovação no Comitê de Ética em Pesquisa (CEP) da Faculdade Frassinetti do Recife (FAFIRE), por meio do parecer nº 6.744.689 e CAAE nº 78472224.6.0000.5586. A coleta de dados seguiu todas as recomendações do Ofício Circular nº 2/2021 da Comissão Nacional de Ética em Pesquisa (CONEP) para pesquisas em ambiente virtual, e todos os participantes assinaram o TCLE. Para preservar o anonimato dos participantes, suas falas foram identificadas com a letra “P” seguida pelo algarismo arábico.

## RESULTADOS

O estudo contou com a participação de 30 estudantes da graduação em Enfermagem da Universidade Federal de Pernambuco (UFPE) e da Universidade de Pernambuco (UPE). Dessa forma, observou-se a participação de 13 graduandos (43%) da UPE e 17 graduandos (57%) da UFPE. O perfil dos discentes foi identificado por meio do formulário sociodemo-gráfico preenchido antes do início das entrevistas, conforme descrito na Tabela 1.

**Tabela 1:** Distribuição do perfil sociodemográfico dos participantes – Recife, PE, Brasil (2024).

Fator avaliado	n	%
Faixa etária		
18 a 21 anos	10	33
22 a 25 anos	15	50
26 a 30 anos	5	17
Sexo		
Feminino	25	83
Masculino	5	17
Raça		
Branca	13	43
Pardo	12	40
Preto	5	17
Universidade		
Universidade Federal de Pernambuco (UFPE)	17	57
Universidade de Pernambuco (UPE)	13	43
Período acadêmico		
6º período	2	7
7º período	2	7
8º período	5	17
9º período	16	53
10º período	4	13
Deslocado, mas já cursou disciplina/módulo de saúde da criança	1	3
Possui graduação anterior?		
Sim	1	3
Não	29	97
Participa de projetos de extensão, atividades e oficinas que atuam com o público infantil?		
Sim	14	47
Não	16	53
Possui pesquisa (PIBIC, PET, outras) voltadas ao público infantil?		
Sim	4	13
Não	26	87

Fonte: Elaborada pelos autores (2024).

Verificou-se que a maioria era do sexo feminino (83%), e apresentava média de idade de 23 anos. Em relação à raça, 44% se autodeclaram brancos, 40% pardos e 17% negros. Além disso, identificou-se que a maioria dos participantes (54%) encontrava-se no 9º período no momento da pesquisa, entre esses apenas 1 (3%) possuía graduação anterior. Do total de participantes, 14 (47%) participaram de projetos de extensão, atividades e oficinas voltados ao público infantil, e 13% participavam de projetos de pesquisa voltados a esse público.

Foi observado pela pesquisadora, a partir da prontidão e da articulação das respostas, maior desenvoltura e domínio da temática por parte dos graduandos de uma determinada IES em relação à outra, pois relataram maior experiência prática com estímulo à produção e à aplicação de materiais educativos lúdicos sobre saúde com o público infantil. Na outra instituição, apenas aqueles que possuíam vivência em projetos de extensão, também envolvendo a produção e a aplicação de materiais educativos lúdicos com esse público, demonstraram maior desenvoltura, articulação e domínio nas respostas.

A partir da análise dos dados e dos discursos dos participantes foram elaboradas quatro categorias: Compreensão sobre as competências e habilidades em educação em saúde; Especificidades requeridas para atuar como educador em saúde com o público infantil; Estratégias e recursos para a educação em saúde com crianças; e Contribuição da graduação em Enfermagem para as ações de educação em saúde.

### Compreensão Sobre as Competências e Habilidades em Educação em Saúde

Ao expressarem seus conhecimentos empíricos para distinguir as competências e habilidades requisitadas ao profissional de saúde, alguns graduandos relacionaram as competências em educação em saúde aos conhecimentos teóricos apreendidos durante o processo formativo, enquanto na definição de habilidade observou-se uma associação com o desempenho prático, como demonstrado no discurso:

*“Competência é o conhecimento que eu detenho, e habilidade é a minha destreza em poder fazer ações educativas. Por exemplo, cada público que a gente assiste exige uma linguagem e uma forma de se portar* (...). *Então, a forma como abordamos a pessoa que está sendo assistida é uma habilidade que desempenhamos nesse âmbito da educação em saúde.” (P13)*

Durante a apreensão dos relatos, também foram identificados alguns graduandos que apresentaram certa dificuldade para expressar seus conhecimentos sobre as competências e habilidades necessárias ao exercício do papel educativo em saúde do enfermeiro, como demonstrado abaixo:

*“Eu nunca pensei nisso, na diferença entre competência e habilidade, mas acredito que habilidade é algo inerente a você, é a capacidade de se comunicar, de você ser compreendido em determinada situação. Já competência deve estar relacionado àquilo que você estudou; ou seja, é ter a competência de passar aquilo que você estudou, passar o seu conhecimento.” (P5)*

*“Para mim, são termos que estão muito intrínsecos e eu não consigo distinguir o que seria uma competência ou que seria uma habilidade nessa questão de educação.” (P3)*

### Especificidades Requeridas para Atuar Como Educador em Saúde com o Público Infantil

Para atuar como educador em saúde com o público infantil, os graduandos trouxeram uma visão relacionada ao planejamento das atividades educativas, as quais devem ter como principal meta a utilização de uma linguagem lúdica e adaptada ao público infantil, o domínio do conhecimento científico sobre a temática a ser abordada e a observação do perfil e o ambiente em que a criança está inserida.

*“Acredito que a criatividade e a linguagem lúdica, adaptada e acessível, pois estamos acostumados a usar muitos termos técnicos; pela força do hábito, muitas vezes a gente não se “policia” em trazer uma linguagem mais acessível* (...). *Se a gente usar o termo técnico, o público não vai conseguir entender a informação que está sendo transmitida e não vai causar o impacto da maneira que desejamos, se fosse adaptado para o público específico.” (P2)*

*“Primeiro, é conseguir perceber as particularidades do público que estamos lidando, compreender como o público infantil está inserido naquele contexto e o que eles gostam, o que é que interessa a eles. Pois, dentro do público infantil, temos uma variedade de interesses, porque a cada faixa etária tem os marcos de desenvolvimento.” (P27)*

*“Requer a sensibilidade, pois você consegue fazer uma leitura corporal das crianças e consegue notar se elas estão construindo uma linha de raciocínio junto a você* (...) *acredito que só assim a educação em saúde terá um efeito positivo, porque, ao entender a importância do assunto, a criança irá incentivar os pais, os responsáveis, os amigos etc.” (P25)*

Alguns graduandos trouxeram um olhar para a aplicação das ações de educação em saúde também no contexto hospitalar, buscando além de envolver a criança, incluir o cuidador, oportunizando cenários adaptativos para esclarecimentos e orientações, de modo prazeroso e criativo, mobilizados pelo sentimento de empatia.

*“*(...)*ter a competência de identificar o público que você irá realizar a ação. Falando particularmente de crianças, eu acredito que seja mais complexo, porque é necessário realizar esse processo de maneira lúdica, para que ela consiga compreender. As crianças já têm um medo cultural de hospital, de enfermeiro, médico etc., então acho difícil fazer essa quebra de pensamento.” (P30)*

### Estratégias e Recursos para a Educação em Saúde com Crianças

Em relação ao uso de estratégias e recursos para viabilizar o processo de ensino-aprendizagem, no contexto infantil, os participantes apontaram como essencial o conhecimento da realidade cotidiana da criança, para que seja possível promover a troca de conhecimentos com o público-alvo, por meio de estratégias lúdicas, do uso de metodologias ativas e de recursos didáticos, os quais, segundo os participantes da pesquisa, garantem a participação ativa.

*“Então, eu acho que desenvolver uma metodologia ativa é a base: que a criança participe, que seja lúdico, que seja dinâmico, trazer algumas coisas do dia a dia para que a criança consiga identificar aquela situação, que seja mais dentro da realidade dela. Eu usei, em alguns momentos, a roda de conversa, onde as crianças podiam falar, opinar sobre a situação, dar exemplos, antes mesmo da gente começar a falar sobre o assunto. Incluir elas nesse diálogo é importante para que elas se interessem mais pelo assunto.” (P1)*

*“Brincadeiras, para fazer com que a criança participe ativamente da atividade* (...) *fazer mais do que só explanar sobre algum assunto; por exemplo, utilizar jogos, encenação teatral, estratégias que coloque a criança como protagonista daquele contexto.” (P16)*

Foi possível observar um favoritismo para o uso de jogos educativos como recurso didático na atualidade, segundo os participantes, o uso desses recursos auxilia na associação entre o conteúdo trabalhado e o cotidiano do público, bem como também se afasta do processo de ensino-aprendizagem tradicional, trazendo a brincadeira como forma de aprendizagem.

*“Lembro do estágio de saúde da criança, pois, quando estávamos na enfermaria, pensamos em trabalhar a temática de higiene pessoal com as crianças internadas. Então, utilizamos a estratégia de jogos, como o jogo da memória, e algumas imagens para eles colorirem, pois enquanto as crianças iam brincando, elas aprendiam sobre a temática.” (P21)*

*“Brincadeiras, competições e figuras. Crianças adoram brincadeiras, principalmente aquelas que envolvem recompensa, e as brincadeiras e competições estimulam elas a participar desse momento de forma espontânea.” (P29)*

Ademais, foi possível notar, como reflexo da pandemia da COVID-19 que alguns graduandos adaptaram suas estratégias lúdicas, anteriormente utilizadas presencialmente, para o contexto on-line, como forma de continuar disseminando informações de saúde ao público infantil.

*“Eu só tive a oportunidade de realizar uma ação educativa para crianças uma vez; no entanto, como foi durante a pandemia, a ação teve que ocorrer de modo on-line. Então, a estratégia que utilizamos foi slides coloridos e lúdicos, e pedimos para eles confeccionarem, previamente, um boneco de garrafa PET para simular as compressões de RCP. Apesar de ser on-line, eu acredito que foi bastante proveitoso o uso dessa estratégia, pois elas interagiram bastante, principalmente no final, quando fizemos a dinâmica de perguntas de verdadeiro ou falso. Infelizmente, eu nunca tive a oportunidade de fazer educação em saúde com crianças pessoalmente, mas acredito que realizar essas estratégias pessoalmente chamaria mais atenção desse público.” (P26)*

### Contribuição da Graduação em Enfermagem para as Ações de Educação em Saúde

Quanto à contribuição da graduação em Enfermagem para o desenvolvimento de competências e habilidades voltadas às ações de educação em saúde, os graduandos destacaram a importância de estar inserido em atividades extracurriculares que estimulam a vivência, principalmente aquelas que são voltadas para o público infantil, pois contribuem para a segurança no desenvolvimento de atividades educativas. Observa-se que, segundo a visão dos participantes, a obtenção de competências e habilidades para lidar com a educação em saúde com o público infantil é pouco abordada nas disciplinas da grade curricular.

*“Na minha experiência, dentro da graduação, eu não vi nada que abordasse sobre educação em saúde para crianças, sobre o lúdico e as estratégias que a gente possa utilizar com esse público, mas em atividades extracurriculares, como projetos de extensão.” (P14)*

*“A graduação em Enfermagem contribui me aproximando de projetos voltados para essa temática, porque, na grade curricular, pelo menos no meu ponto de vista, trata muito sobre as doenças e afecções das crianças, sendo muito focado para o lado assistencial. E, quando temos o auxílio de projetos de extensão ou ligas acadêmicas, nós conseguimos desenvolver melhor essas habilidades. Além disso, as monitorias dadas pelos próprios alunos nos permite perceber como é planejar e executar uma aula, além de nos auxiliar nesse processo de repassar o conteúdo que aprendemos.” (P27)*

Ademais, os graduandos também trouxeram a necessidade de ajustes na grade curricular visando à melhoria do desenvolvimento destas competências e habilidades. Entre os pontos elencados para aprimoramento, destacam-se: a redução do foco nas práticas de assistência hospitalar; a inserção de aulas voltadas para estratégias e metodologias ativas que possam ser utilizadas em educação em saúde; discussões sobre a educação popular com a inclusão do público infantil; bem como o estímulo ao desenvolvimento de ações educativas com crianças, como descrito nas falas abaixo:

*“A graduação foca muito na parte clínica e esquece que a educação em saúde vai desde a atenção básica até a alta complexidade. E, apesar dessa importância, durante a graduação eu não me recordo de ter aulas sobre as estratégias de educação em saúde; então, o que eu aprendi foi durante a prática, através das tentativas e erros, e fui aprimorando para os diferentes contextos.” (P22)*

## DISCUSSÃO

Ao estimular o pensamento crítico sobre as competências e habilidades em educação em saúde, os graduandos puderam apresentar suas compreensões a respeito da temática. No que se refere às competências, nota-se a relação intrínseca com a obtenção de conhecimento científico e com a capacidade de transformá-lo em conteúdo acessível aos diferentes públicos, o que exige não apenas competências, mas também habilidades educativas. Assim, pode-se afirmar que as competências englobam um conjunto de conhecimentos, atitudes, motivações, autopercepções e habilidades que tornam possível a realização da ação educativa^(17)^.

Apesar de compreender o conceito de educação em saúde, houve certa dificuldade de alguns discentes para correlacionar este entendimento com as competências necessárias ao profissional enfermeiro no âmbito da promoção da saúde. Seguindo uma concepção alinhada à literatura nacional e às DCNs de Enfermagem, entende-se por competência, no âmbito da formação, a mobilização de conhecimentos, habilidades, atitudes e valores, os quais possibilitam ao egresso adotar uma postura de liderança, comunicação, tomada de decisões e atuação como facilitador em ações educativas^(3)^.

Embora se ratifique a visão do enfermeiro como educador em saúde, nem sempre se evidencia a compreensão sobre as competências que esse profissional deve desenvolver para que tal papel se consolide como ferramenta de trabalho. Com base nos achados da literatura, as competências devem estar relacionadas ao julgamento clínico, à comunicação, ao cuidado centrado na pessoa, à equidade, à inclusão, à ética, à prática baseada em evidências, às políticas de saúde e aos determinantes sociais da saúde^(18)^.

Em relação às habilidades educativas, a compreensão estendeu-se às capacidades práticas, à ação e ao momento educativo propriamente dito, sendo descrita como a capacidade de transformar o conhecimento científico em conhecimento popular por meio de diferentes metodologias. Ao refletir sobre as habilidades requisitadas ao profissional enfermeiro no exercício de ações educativas, observa-se que elas se referem a dimensões práticas, cognitivas e socioemocionais que orientam a mobilização das competências no trabalho em saúde. Ao incentivar o desenvolvimento de tais habilidades durante a graduação, possibilita-se ao futuro profissional um melhor desempenho assistencial e educativo^(19,20)^.

Além disso, foi possível notar que a habilidade de comunicação foi a mais citada entre os participantes da pesquisa. Esse entendimento é reforçado pela literatura, em que Daza-Betancourt et al.^(21)^ destacaram que a habilidade de comunicação assume papel central, pois dentre outras a serem desenvolvidas pelo enfermeiro, torna-se essencial por oportunizar a criação de vínculos, confiança, empatia, além de facilitar a construção de um ambiente propício à troca de informações.

Em relação à categoria “Especificidades requeridas para atuação do profissional enfermeiro como educador em saúde com o público infantil”, foi possível notar a preocupação dos graduandos quanto à promoção da saúde infantil por meio de atividades educativas lúdicas, acessíveis, adaptadas ao público e à realidade em que as crianças estão inseridas. Ao pensar em educação em saúde, o ambiente escolar destaca-se por possibilitar ação intersetorial entre saúde e educação. Dentre os profissionais da saúde, o enfermeiro assume o papel de protagonista como agente mobilizador e articulador das ações educativas, pois ele utiliza das suas competências para promover a prevenção e proteção contra agravos, visando mitigar vulnerabilidades e contribuir para o desenvolvimento integral de crianças e adolescentes^(22)^.

Ao considerar o contexto social, econômico e de saúde em que o público está inserido, é possível planejar uma ação que simule o cotidiano dentro daquela população, configurandose como fator estimulante para que a criança se torne multiplicadora de conhecimentos em saúde na comunidade. Embora o lúdico atue como facilitador do processo de ensinoaprendizagem, é necessário ir além de linguagem acessível e afastada de termos técnicos; deve-se considerar o contexto de vida da criança, suas opiniões, interesses e raciocínios próprios, para que seja possível sensibilizá-la, motivando-a por meio da relação das suas vivências com o conteúdo abordado^(23)^.

Ao planejar ações de educação em saúde, o facilitador, além do conhecimento científico sobre o tema a ser abordado, deve utilizar de metodologias que se constituem pontes para a construção do conhecimento junto ao público-alvo, bem como prever e manejar possíveis imprevistos durante a ação educativa.

Em consonância com os achados da pesquisa, compreendese que, para exercer atividade educativa no cenário infantil, é necessário planejar o momento educativo considerando local, público-alvo, duração da atividade e conteúdo, com o intuito de promover o conhecimento por meio do estabelecimento de relação de confiança^(24)^. Além disso, ao definir que o público da atividade educativa seja composto por crianças, o educador em saúde deve considerar as diferentes abordagens correspondentes aos distintos estágios do desenvolvimento infantil, pois o insucesso de algumas ações educativas está relacionado à generalização de materiais e métodos para diferentes faixas etárias, em desacordo com a literatura, que preconiza que a implementação de atividades educativas na infância deve considerar os estágios de desenvolvimento da criança^(25)^.

Para o desenvolvimento do processo de ensino-aprendizagem, no contexto infantil, os graduandos destacaram como essencial a articulação da ação educativa com o cotidiano da criança, utilizando de estratégias como teatro de fantoches, encenação teatral, contação de histórias, paródias, vídeos educativos, desenhos, brinquedos, musicoterapia, entre outras, pois tais estratégias lúdicas garantem participação ativa e viabilizam a troca de conhecimento com o público-alvo. Nessa perspectiva, a literatura aponta que essas estratégias apresentam potencial de aprendizagem, principalmente porque a brincadeira permite a simulação de cenários e contextos, onde as crianças podem explorar, tirar dúvidas e construir um repertório sobre o assunto abordado^(26)^.

Entre as estratégias educativas referidas, além das que simulam o cotidiano da criança, destacaram-se estratégias que estimulam o protagonismo infantil, como a construção de rodas de conversa, nas quais as crianças são incentivadas a falar, expressar opiniões e apresentar exemplos. Ao utilizar estratégias que promovem o protagonismo infantil, evidencia-se o sucesso da atividade educativa, pois, embora o uso de materiais lúdicos seja amplamente difundido, o sucesso da intervenção em saúde reside nas relações estabelecidas durante o processo, não se restringindo ao uso de objetos no momento educativo^(23)^.

Ademais, dentre os recursos utilizados para garantir a participação ativa do público infantil, os graduandos destacaram o uso de jogos educativos, tanto tradicionais quanto digitais, com ênfase no desenvolvimento de tecnologias educativas virtuais. Observou-se predileção pelo desenvolvimento e aplicação de jogos educativos digitais com crianças, uma vez que esses estimulam a aprendizagem por meio da valorização das experiências prévias, favorecem o diálogo entre os facilitadores e o público-alvo e contribuem para o desenvolvimento de aspectos emocionais, cognitivos e motores, além de se distanciarem das metodologias tradicionais de ensino^(27)^.

Também se evidenciou, nos resultados da pesquisa, o impacto da pandemia da COVID-19 sobre as ações de promoção da saúde com o público infantil, tornando necessária a reformulação das metodologias tradicionalmente utilizadas para viabilizar a continuidade das ações educativas. Nesse contexto, introduziu-se o uso das Tecnologias de Informação e Comunicação (TICs), que compreendem recursos eletrônicos que permitem comunicar, interagir e aprender, tais como *softwares* educacionais, vídeos educativos, jogos, *sites*, imagens, possibilitando a promoção da saúde para além das barreiras físicas^(28)^.

Nos achados da pesquisa, evidenciou-se uma limitação na expressão da compreensão sobre competências e habilidades, bem como também sobre as estratégias e os recursos a serem utilizados em atividades educativas com o público infantil, entre os graduandos que não apresentavam experiência extensionista ou atuação prática. A inserção no cenário escolar, a escuta das demandas dos escolares e a integração com os professores e gestores contribuem para uma maior segurança e desenvolvimento de *expertise* na condução de ações educativas, ressaltando a importância do tripé universitário.

A integração entre o ensino, pesquisa e extensão promovida dentro do ambiente acadêmico foi enfatizada como principal contribuição para o desenvolvimento de competências e habilidades voltadas às ações de educação em saúde, uma vez que permite a inserção de graduandos em cenários teórico-práticos, favorecendo uma visão multidimensional, desenvolvimento de um olhar clínico crítico e contextualização do conhecimento para a transformação social^(29)^. Além disso, os graduandos referiram a importância da monitoria acadêmica como estratégia para aprimorar os conhecimentos adquiridos na disciplina e desenvolver habilidades de ensino-aprendizagem, visto que a monitoria tem como propósito esclarecer dúvidas referentes à área de conhecimento, auxiliar no aprofundamento teórico e reforçar habilidades práticas apreendidas durante a disciplina^(30)^.

Salienta-se a insatisfação dos graduandos quanto à grade curricular, os quais ressaltam maior proximidade das disciplinas com doenças e afecções e afastamento da perspectiva da educação popular em saúde, especialmente relacionada ao público infantil. Evidenciou-se nos discursos a necessidade de ajustes curriculares, considerando que, para melhor capacitar futuros enfermeiros, o currículo da graduação deve estar alinhado às transformações na prestação de cuidados em saúde para os diferentes públicos. Nesse sentido, o ambiente acadêmico configura-se como o espaço de transformação e alinhamento às necessidades atuais e futuras no âmbito da saúde^(17)^.

Embora o desenvolvimento das competências e habilidades do enfermeiro ocorra de maneira gradual, a partir do acúmulo de experiências com diferentes públicos e da ressignificação dos conhecimentos frente a novas situações, os discentes relataram dificuldade e insegurança para promover ações educativas ao público infantil, devido ao maior enfoque da graduação no público adulto e idoso. Nesse sentido, para reduzir tal insegurança, os graduandos devem ser estimulados durante a formação, ao desenvolvimento da autonomia na aquisição de conhecimentos em saúde, ao aprimoramento da comunicação, ao fortalecimento das relações interpessoais e à resolutividade^(31)^.

Para que isto ocorra, as IES podem adotar metodologias ativas de aprendizagem na formação profissional, visto que essas simulam ou inserem o acadêmico no campo de atuação com o objetivo de oportunizar a apreensão de habilidades em saúde^(32)^. Entre tais estratégias, destaca-se a utilização de tecnologias educativas digitais, que favorecem o desenvolvimento cognitivo e atitudinal necessário ao contexto de promoção da saúde, além de diversificar o processo de ensino-aprendizagem^(30)^.

Embora este estudo tenha contribuído na compreensão das competências e habilidades necessárias aos graduandos de Enfermagem para atuarem como educadores em saúde na atenção à saúde da criança, observou-se que a inclusão apenas de universidades públicas e de um único estado configura limitação quanto ao aprofundamento da temática. Ademais, identificaram-se as lacunas encontradas na literatura sobre a conceituação de competências em educação em saúde e de habilidades educativas no contexto da Enfermagem, o que restringiu uma discussão mais específica e comparativa dos achados. Portanto, sugere-se que estudos futuros ampliem a compreensão desses conceitos, abrangendo maior quantidade de IES, incluindo as instituições privadas, que concentram uma parcela significativa dos graduandos de Enfermagem.

## CONCLUSÃO

A percepção dos graduandos de Enfermagem sobre as competências e habilidades necessárias para a educação em saúde no contexto infantil evidencia a existência de um déficit na formação curricular, especialmente no que se refere à preparação para atuar como educadores em saúde de crianças. Embora demonstrem compreender a importância de articular o conteúdo científico com metodologias lúdicas para transformá-lo em conhecimento acessível e significativo, os discentes revelaram insegurança e dificuldade em aplicar essas práticas, o que é atribuído à escassa vivência com o público infantil durante a graduação.

Entretanto, para os graduandos com vivência teórico-prática em contexto escolar, a competência pode ser definida como a articulação integrada entre conhecimentos, habilidades e atitudes, requerida para que o profissional mobilize o que sabe (conhecimento) e saiba como fazer (habilidade), de modo contextualizado e adequado ao público e à situação vivenciada. Destaca-se, ainda, a importância da sensibilidade do discente para reconhecer e valorizar as especificidades de cada público, considerando suas reais necessidades e o compromisso de propiciar uma arena lúdica e criativa, capaz de motivar e estimular a participação das crianças.

Nesse cenário, destacam-se as atividades extensionistas, os grupos de pesquisa e a monitoria acadêmica como espaços privilegiados para o desenvolvimento dessas competências, uma vez que promovem a inserção dos estudantes em contextos reais e estimulam a integração entre teoria e prática. Assim, tais experiências complementam as lacunas curriculares e contribuem para a formação de enfermeiros mais preparados para atuar como educadores em saúde no cuidado à criança.

## Data Availability

Todo o conjunto de dados que dá suporte aos resultados deste estudo está disponível mediante solicitação ao autor correspondente.

# Translation: Competencias y habilidades de los estudiantes de enfermería como educadores en el cuidado de la salud infantil

**DOI:** 10.1590/1980-220X-REEUSP-2025-0385es

**Authors:** Thays Mylena Lima da Silva, Débora Maria Santana da Silva, Gracielly Karine Tavares Souza, Cândida Maria Rodrigues dos Santos, Eliane Maria Ribeiro de Vasconcelos, Estela Maria Leite Meirelles Monteiro

**Affiliations:** 1 Universidade Federal de Pernambuco, Recife, PE, Brasil.

**Keywords:** Educación Basada en Competencias, Habilidades Sociales, Enfermería, Salud Infantil

## Abstract

**Objetivo:**

Analizar la percepción de los estudiantes de enfermería de pregrado con respecto a las competencias y habilidades necesarias para impartir educación para la salud en el contexto de la salud infantil.

**Método:**

El estudio se caracteriza por ser exploratorio, descriptivo y cualitativo. Se desarrolló en un entorno virtual a través de entrevistas en *Google Meet,* entre marzo y mayo de 2024. La población estudiada estuvo compuesta por 30 estudiantes de enfermería de dos universidades públicas de Pernambuco. Las entrevistas tuvieron una duración máxima de 30 minutos e incluyeron seis preguntas abiertas. Las transcripciones se realizaron en la plataforma *Transkryptor,* unificadas en un solo documento y analizadas por *Software NVivo*.

**Resultados:**

A partir del análisis de datos, se desarrollaron cuatro categorías: Comprensión de las competencias y habilidades necesarias en la educación para la salud; Requisitos específicos para trabajar como educador de salud con niños; Estrategias y recursos para la educación para la salud con niños; y Contribución de un título de enfermería a las iniciativas de educación para la salud.

**Conclusión:**

La percepción que tienen los estudiantes de enfermería sobre las competencias y habilidades necesarias para la educación sanitaria en el contexto de la infancia revela una deficiencia en su plan de estudios, especialmente en lo que respecta a la preparación para trabajar como educadores sanitarios para niños.

## INTRODUCCIÓN

La educación en salud, basada en los principios de la educación popular en salud, surge con el compromiso de asegurar su aplicación en la práctica cotidiana de la atención de salud, con énfasis en su promoción, en la contextualización y en la interrelación entre los conocimientos científicos y populares, considerando los determinantes sociales y las culturas locales para, de manera efectiva, promover el cuidado, la equidad y la participación social^(1)^.

En este sentido, a lo largo de los años, la educación en salud ha incorporado diversas metodologías y tecnologías de enseñanza, con el fin de consolidarse en el ámbito de la salud y la pedagogía. En el ámbito de la salud, su objetivo principal es la promoción de la salud y el cambio de hábitos de vida en los diferentes públicos y contextos de salud, así como proporcionar la formación de profesionales críticos y competentes en lo que se refiere al ejercicio de la profesión y al posicionamiento frente a los males sociales, a través de la educación, ratificando las políticas de salud y las necesidades de la población^(2)^.

Las Directrices Curriculares Nacionales (DCN) establecen que la formación del enfermero debe basarse en principios humanistas, científicos y ético-políticos, integrando la enseñanza, la investigación y la extensión, y garantizando experiencias que favorezcan el desarrollo de competencias técnico-científicas y socioculturales para la atención integral^(3)^.

Desde esta perspectiva, la educación popular en salud se configura como un eje transversal y metodológico en la formación, ya que estimula la praxis educativa problematizadora y el compromiso social con la equidad, la ciudadanía y la transformación de las realidades locales. De este modo, la licenciatura, en especial los cursos del área de la salud, se convierte en una herramienta importante para el desarrollo de habilidades y la consolidación de conocimientos relacionados con las prácticas educativas en salud^(4)^.

Según la literatura, las competencias pueden definirse como el conjunto de conocimientos, habilidades y actitudes necesarios para el desarrollo de tareas y para la actuación en la resolución de problemas, lo que capacita al individuo para ejercer una determinada profesión^(5)^. En consonancia, las habilidades pueden definirse como herramientas que potencian la capacidad técnica en un determinado ámbito de actuación profesional^(6)^.

Sin embargo, en lo que respecta al desarrollo de competencias y habilidades para el cuidado y la promoción de la salud de la población infantil, el enfermero o el estudiante de Enfermería, en ocasiones, experimenta un sentimiento de inseguridad, ya que el manejo y la comunicación en salud con este público deben individualizarse de acuerdo con el grupo de edad y la etapa de desarrollo^(7)^. Además, el enfermero educador en salud infantil debe desarrollar habilidades comunicativas, lúdicas y dialógicas que favorezcan el protagonismo de estos niños como agentes activos del aprendizaje sobre autocuidado y promoción de la salud^(8)^. En este sentido, para que la educación en salud en el grupo de edad infantil sea efectiva, es necesario desarrollar competencias y habilidades desde la licenciatura^(9)^.

Así, al pensar en la educación en salud para la población infantil, es necesario comprender que el desarrollo de este público objetivo es dinámico y que este no se configura como mero receptor de información, sino como individuo capaz de desarrollar autonomía a través del lenguaje y de la experimentación en el mundo que lo rodea^(7)^. Además, los hallazgos indican una definición insuficiente, una contextualización nacional limitada y un desajuste curricular de las competencias y habilidades que los estudiantes de grado de Enfermería necesitan para actuar como educadores en salud en la atención infantil —tanto por la ausencia de evidencia brasileña reciente en salud escolar como por divergencias internacionales y por un desajuste entre la formación y la práctica^(10,11,12)^. En consonancia, este estudio tiene como objetivo analizar la percepción de los estudiantes de enfermería sobre las competencias y habilidades necesarias para la educación en salud en el contexto de la salud infantil.

## MÉTODO

### Diseño del Estudio

Se trata de un estudio cualitativo, exploratorio y descriptivo, basado en entrevistas semiestructuradas. Los datos se analizaron mediante el análisis de contenido temático, según la propuesta de Bardin^(13)^. El informe del estudio siguió las recomendaciones de los *Consolidated Criteria for Reporting Qualitative Research* (COREQ)^(14)^, con el objetivo de garantizar la transparencia y la exhaustividad en la presentación de la investigación cualitativa. Se contemplaron todos los ítems aplicables de acuerdo con la naturaleza y los objetivos de la investigación.

### Lugar

El estudio se llevó a cabo en un entorno virtual, a través de la plataforma *Google Meet*, con el fin de flexibilizar los horarios de las entrevistas, ya que la mayoría de las clases de los cursos de licenciatura en Enfermería se imparten a tiempo completo.

### Población

La población estuvo compuesta por estudiantes de licenciatura del curso de Enfermería de dos universidades públicas del estado de Pernambuco (Brasil), quienes expresaron sus opiniones sobre el tema abordado.

### Criterios de Selección

Se incluyeron en el estudio estudiantes de la carrera de Enfermería matriculados regularmente en Instituciones de Educación Superior (IES) que, al momento de la recopilación, ya hubieran cursado el módulo o la asignatura de Salud Infantil.

### Recolección de Datos

La recolección de datos se llevó a cabo de marzo a mayo de 2024. La selección de los participantes se realizó mediante la técnica de muestreo *snowball*, que consiste en utilizar a un individuo de referencia, denominado semilla, el cual indica a un participante que cumpla con los criterios de participación en la investigación^(12)^. Para captar al individuo de referencia, se envió una carta de invitación, que contenía información sobre los objetivos del estudio y sobre la participación. Esta carta se envió por correo electrónico a un representante de cada clase que cumplía con los criterios de selección, facilitados por la secretaría de las IES participantes. Los representantes aceptaron participar e indicaron a un nuevo miembro; los demás contactos se gestionaron a través de las redes sociales (*Instagram* y *WhatsApp*).

A partir del contacto inicial con el participante, se programaba la entrevista, de manera individual, considerando el mejor día y horario. En el momento de la entrevista, se leía, junto con el participante, el Término de Consentimiento Libre e Informado (TCLE) y el Término de Autorización de Imagen y Testimonio. Tras la aceptación formal para participar en el estudio, se le pedía al estudiante que respondiera un formulario, a través de *Google Forms*, que contenía 11 preguntas objetivas con datos sociodemográficos, con el fin de trazar el perfil de los participantes. La investigadora también utilizó un guion de observación de datos subjetivos, captados principalmente por el tono de voz y el tiempo de respuesta, lo que requirió sensibilidad para analizar el interés, la articulación, el dominio y la seguridad.

Durante la recolección de datos en el entorno virtual, se produjo, en una sola entrevista, una interrupción frecuente debido a la inestabilidad de la *Internet*, por lo que se acordó la necesidad de reprogramarla, cuando fue posible la nueva participación. De este modo, no hubo pérdida de la muestra.

A continuación, se iniciaba la entrevista con la grabación de la pantalla, siendo opcional para el participante la activación de la *cámara web.* Cabe destacar que las entrevistas tuvieron una duración máxima de 30 minutos, en las que se realizaron seis preguntas subjetivas basadas en los objetivos de la investigación, a saber:

“*¿Qué entiende usted por competencias en educación en salud?”; “¿Cómo explicaría la diferencia entre competencias y habilidades?”; “En su opinión, ¿cuáles son las competencias necesarias para desarrollar acciones de educación en salud dirigidas al público infantil?”; “Basándose en su experiencia, ¿qué estrategias utiliza(ba) para desarrollar acciones de educación en salud para niños? ¿Alguna de ellas la aprendió durante la carrera?”; “Si fuera a realizar una acción educativa para niños, ¿qué materiales/herramientas utilizaría?”; “En su opinión, ¿cómo contribuye la carrera de Enfermería al desarrollo de estas competencias y habilidades?”*.

Para comprobar la claridad del guion de la entrevista, se elaboró un estudio piloto con tres participantes, con el fin de observar la comprensibilidad de las preguntas. Como no fue necesario revisar el guion, los participantes de la aplicación piloto se incluyeron en la muestra.

La recolección de datos se interrumpió cuando se constató la saturación de respuestas durante las entrevistas, tal como lo recomienda el enfoque metodológico, en el que, en las investigaciones cualitativas, es más importante abarcar las dimensiones del tema elegido que el número de participantes^(15)^.

### Análisis y Tratamiento de los Datos

Los relatos de los participantes, en formato de audio, se transcribieron íntegramente con la ayuda de la plataforma *Transkriptor.* Sin embargo, fue necesario revisar el material para garantizar la preservación del contenido literal de los discursos. Tras la organización de los datos recopilados, se inició el análisis mediante el método de Análisis de Contenido, cuyo objetivo es categorizar e interpretar los hallazgos del estudio. Según Bardin^(13)^, esta práctica puede desarrollarse en tres fases: el preanálisis; la exploración del material; y el tratamiento de los resultados. En el preanálisis, la investigadora agregó los relatos de los participantes a los registros de datos subjetivos percibidos durante la entrevista, en la que cada pregunta correspondió, en un primer momento, a una variable, y posteriormente, los datos se unificaron en un documento. A continuación, la etapa de exploración del material se llevó a cabo con la ayuda del *software NVivo* ^(16)^, con el fin de facilitar la identificación de las unidades que componen la descripción de las características evidenciadas del contenido, contribuyendo a la clasificación y la agregación en la definición de las categorías^(15)^. Por último, se realizó un análisis inductivo de las narrativas recopiladas y organizadas mediante la similitud semántica^(15)^.

### Aspectos Éticos

Se respetaron todos los aspectos éticos descritos en la Resolución n.º 466/2012 del Consejo Nacional de Salud (CNS) y con la aprobación del Comité de Ética en Investigación (CEP) de la Facultad Frassinetti de Recife (FAFIRE), mediante el dictamen n.º 6.744.689 y el CAAE n.º 78472224.6.0000.5586. La recolección de datos siguió todas las recomendaciones de la Circular n.º 2/2021 de la Comisión Nacional de Ética en Investigación (CONEP) para investigaciones en entornos virtuales, y todos los participantes firmaron el TCLE. Para preservar el anonimato de los participantes, sus declaraciones se identificaron con la letra “P” seguida de un número arábigo.

## RESULTADOS

El estudio contó con la participación de 30 estudiantes de la carrera de Enfermería de la Universidad Federal de Pernambuco (UFPE) y de la Universidad de Pernambuco (UPE). Así, se observó la participación de 13 estudiantes (43 %) de la UPE y 17 estudiantes (57 %) de la UFPE. El perfil de los estudiantes se identificó mediante el formulario sociodemográfico completado antes del inicio de las entrevistas, tal como se describe en la Tabla 1.

**Tabla 1:** Distribución del perfil sociodemográfico de los participantes – Recife, PE, Brasil (2024).

Factor evaluado	n	%
Grupos de edad		
18 a 21 anos	10	33
22 a 25 anos	15	50
26 a 30 anos	5	17
Sexo		
Femenino	25	83
Masculino	5	17
Raza		
Blanca	13	43
Parda	12	40
Negra	5	17
Universidad		
Universidad Federal de Pernambuco (UFPE)	17	57
Universidad de Pernambuco (UPE)	13	43
Semestre académico		
6º período	2	7
7º período	2	7
8º período	5	17
9º período	16	53
10º período	4	13
No está matriculado, pero ya ha cursado la asignatura o el módulo sobre salud infantil	1	3
¿Tiene una titulación previa?		
Si	1	3
No	29	97
¿Participas en proyectos de extensión, actividades y talleres dirigidos al público infantil?		
Si	14	47
No	16	53
¿Realiza investigaciones (PIBIC, PET, otras) dirigidas al público infantil?		
Si	4	13
No	26	87

Fuente: Elaborada por los autores (2024).

Se verificó que la mayoría eran mujeres (83 %) y presentaban una edad promedio de 23 años. En cuanto a la raza, el 44 % se autodeclaró blanco, el 40 % mestizo y el 17 % negro. Además, se identificó que la mayoría de los participantes (54 %) se encontraba en el noveno período en el momento de la investigación; de ellos, solo 1 (3 %) poseía un título de grado anterior. Del total de participantes, 14 (47 %) participaron en proyectos de extensión, actividades y talleres dirigidos al público infantil, y el 13 % participaba en proyectos de investigación dirigidos a este público.

La investigadora observó, a partir de la rapidez y la coherencia de las respuestas, una mayor soltura y dominio del tema por parte de los estudiantes de una determinada institución de educación superior en comparación con la otra, ya que estos relataron una mayor experiencia práctica en el fomento de la producción y la aplicación de materiales educativos lúdicos sobre salud dirigidos al público infantil. En la otra institución, solo aquellos que contaban con experiencia en proyectos de extensión, que también implicaban la producción y aplicación de materiales educativos lúdicos con este público, demostraron mayor desenvoltura, articulación y dominio en las respuestas.

A partir del análisis de los datos y de los discursos de los participantes se elaboraron cuatro categorías: Comprensión de las competencias y habilidades en educación en salud; Especificidades requeridas para actuar como educador en salud con el público infantil; Estrategias y recursos para la educación en salud con niños; y Contribución de la carrera de Enfermería a las acciones de educación en salud.

## Comprensión de las Competencias y Habilidades en Educación para la Salud

Al expresar sus conocimientos empíricos para distinguir las competencias y habilidades requeridas al profesional de la salud, algunos estudiantes de pregrado relacionaron las competencias en educación para la salud con los conocimientos teóricos adquiridos durante el proceso formativo, mientras que en la definición de habilidad se observó una asociación con el desempeño práctico, como se demuestra en el discurso:

*“La competencia es el conocimiento que poseo, y la habilidad es mi destreza para llevar a cabo acciones educativas. Por ejemplo, cada público al que atendemos exige un lenguaje y una forma de comportarse* (...). *Entonces, la forma en que abordamos a la persona que está siendo atendida es una habilidad que desempeñamos en este ámbito de la educación en salud.” (P13)*

Durante el análisis de los relatos, también se identificaron algunos estudiantes de pregrado que presentaron cierta dificultad para expresar sus conocimientos sobre las competencias y habilidades necesarias para el ejercicio del papel educativo en salud del enfermero, como se muestra a continuación:

*“Nunca había pensado en eso, en la diferencia entre competencia y habilidad, pero creo que la habilidad es algo inherente a uno mismo, es la capacidad de comunicarse, de ser comprendido en una situación determinada. La competencia, en cambio, debe estar relacionada con lo que uno ha estudiado; es decir, es tener la competencia para transmitir lo que uno ha estudiado, transmitir su conocimiento.” (P5)*

*“Para mí, son términos muy intrínsecos y no logro distinguir qué sería una competencia o qué sería una habilidad en esta cuestión de la educación.” (P3)*

## Especificidades Requeridas para Actuar Como Educador en Salud con el Público Infantil

Para ejercer como educador en salud con el público infantil, los estudiantes de pregrado aportaron una visión relacionada con la planificación de las actividades educativas, las cuales deben tener como objetivo principal el uso de un lenguaje lúdico y adaptado al público infantil, el dominio del conocimiento científico sobre la temática a abordar y la observación del perfil y el entorno en el que se encuentra la niña o el niño.

*“Creo que la creatividad y el lenguaje lúdico, adaptado y accesible, ya que estamos acostumbrados a usar muchos términos técnicos; por la fuerza de la costumbre, muchas veces no nos “vigilamos” para usar un lenguaje más accesible* (...).

*Si usamos el término técnico, el público no podrá entender la información que se está transmitiendo y no causará el impacto que deseamos, si se adaptara al público específico.” (P2)*

*“En primer lugar, se trata de percibir las particularidades del público con el que estamos tratando, comprender cómo el público infantil se inserta en ese contexto y qué les gusta, qué les interesa. Porque, dentro del público infantil, tenemos una variedad de intereses, ya que cada grupo de edad tiene sus propios hitos de desarrollo.” (P27)*

*“Requiere sensibilidad, ya que puedes leer el lenguaje corporal de los niños y notar si están construyendo una línea de razonamiento contigo* (...) *creo que solo así la educación en salud tendrá un efecto positivo, porque, al entender la importancia del tema, el niño animará a los padres, a los responsables, a los amigos, etc.” (P25)*

Algunos estudiantes de pregrado abordaron la aplicación de las acciones de educación en salud también en el contexto hospitalario, buscando, además de involucrar al niño, incluir al cuidador, creando escenarios adaptativos para aclaraciones y orientaciones, de manera placentera y creativa, impulsados por el sentimiento de empatía.

*“*(...)*tener la competencia para identificar al público al que se dirigirá la acción. Hablando específicamente de niños, creo que es más complejo, porque es necesario llevar a cabo este proceso de manera lúdica, para que puedan comprenderlo. Los niños ya tienen un miedo cultural al hospital, al enfermero, al médico, etc., así que me parece difícil romper con ese pensamiento.” (P30)*

## Estrategias y Recursos para la Educación en Salud con Niños

En relación con el uso de estrategias y recursos para viabilizar el proceso de enseñanza-aprendizaje, en el contexto infantil, los participantes señalaron como esencial el conocimiento de la realidad cotidiana del niño, para que sea posible promover el intercambio de conocimientos con el público objetivo, mediante estrategias lúdicas, el uso de metodologías activas y de recursos didácticos, los cuales, según los participantes de la investigación, garantizan la participación activa.

*“Entonces, creo que desarrollar una metodología activa es la base: que el niño participe, que sea lúdico, que sea dinámico, traer algunas cosas del día a día para que el niño pueda identificar esa situación, que esté más dentro de su realidad. Usé, en algunos momentos, el círculo de conversación, donde los niños podían hablar, opinar sobre la situación, dar ejemplos, incluso antes de que empezáramos a hablar sobre el tema. Incluirlas en este diálogo es importante para que se interesen más por el tema.” (P1)*

*“Juegos, para lograr que el niño participe activamente en la actividad* (...) *hacer algo más que solo explicar un tema; por ejemplo, utilizar juegos, representaciones teatrales, estrategias que sitúen al niño como protagonista de ese contexto.” (P16)*

Se observó una preferencia por el uso de juegos educativos como recurso didáctico en la actualidad; según los participantes, el uso de estos recursos ayuda a asociar el contenido trabajado con la vida cotidiana del público, además de alejarse del proceso tradicional de enseñanza-aprendizaje, incorporando el juego como forma de aprendizaje.

*“Recuerdo las prácticas de salud infantil, pues, cuando estábamos en la enfermería, pensamos en trabajar el tema de la higiene personal con los niños hospitalizados. Entonces, utilizamos la estrategia de los juegos, como el juego de la memoria, y algunas imágenes para que las colorearan, ya que mientras los niños jugaban, aprendían sobre el tema.” (P21)*

*“Juegos, competencias y figuras. A los niños les encantan los juegos, sobre todo aquellos que implican una recompensa, y los juegos y las competencias los estimulan a participar en ese momento de manera espontánea.” (P29)*

Además, se pudo observar, como reflejo de la pandemia de COVID-19, que algunos estudiantes de pregrado adaptaron sus estrategias lúdicas, que antes se utilizaban de manera presencial, al contexto en línea, como forma de seguir difundiendo información de salud al público infantil.

*“Solo tuve la oportunidad de realizar una actividad educativa para niños una vez; sin embargo, como fue durante la pandemia, la actividad tuvo que llevarse a cabo en línea. Entonces, la estrategia que utilizamos fue diapositivas coloridas y lúdicas, y les pedimos que confeccionaran, previamente, un muñeco con una botella de PET para simular las compresiones de la RCP. A pesar de ser en línea, creo que el uso de esta estrategia fue bastante provechoso, ya que interactuaron mucho, sobre todo al final, cuando hicimos la actividad de preguntas de verdadero o falso. Lamentablemente, nunca tuve la oportunidad de impartir educación en salud a niños en persona, pero creo que llevar a cabo estas estrategias en persona llamaría más la atención de este público.” (P26)*

## Contribución de la Carrera de Enfermería a las Acciones de Educación en Salud

En cuanto a la contribución de la carrera de Enfermería al desarrollo de competencias y habilidades orientadas a las acciones de educación en salud, los estudiantes destacaron la importancia de participar en actividades extracurriculares que estimulen la experiencia práctica, sobre todo aquellas dirigidas al público infantil, ya que contribuyen a la seguridad en el desarrollo de actividades educativas. Se observa que, según la opinión de los participantes, la adquisición de competencias y habilidades para abordar la educación en salud con el público infantil apenas se aborda en las asignaturas del plan de estudios.

*“En mi experiencia, dentro de la carrera, no vi nada que abordara la educación en salud para niños, sobre el juego y las estrategias que podemos utilizar con este público, sino en actividades extracurriculares, como proyectos de extensión.” (P14)*

*“La carrera de Enfermería me ayuda a acercarme a proyectos centrados en esta temática, porque, en el plan de estudios, al menos desde mi punto de vista, se trata mucho sobre las enfermedades y afecciones de los niños, centrándose mucho en el aspecto asistencial. Y, cuando contamos con la ayuda de proyectos de extensión o ligas académicas, logramos desarrollar mejor estas habilidades. Además, las tutorías impartidas por los propios estudiantes nos permiten comprender cómo se planifica y ejecuta una clase, además de ayudarnos en este proceso de transmitir el contenido que aprendemos.” (P27)*

Asimismo, los estudiantes de pregrado también señalaron la necesidad de ajustes en el plan de estudios con el fin de mejorar el desarrollo de estas competencias y habilidades. Entre los puntos señalados para su mejora, destacan: la reducción del enfoque en las prácticas de asistencia hospitalaria; la incorporación de clases orientadas a estrategias y metodologías activas que puedan utilizarse en la educación en salud; debates sobre la educación popular con la inclusión del público infantil; así como el estímulo al desarrollo de acciones educativas con niños, tal como se describe en los siguientes comentarios:

*“La carrera se centra mucho en la parte clínica y olvida que la educación en salud abarca desde la atención básica hasta la alta complejidad. Y, a pesar de esta importancia, durante la carrera no recuerdo haber tenido clases sobre estrategias de educación en salud; por lo tanto, lo que aprendí fue durante la práctica, a través de intentos y errores, y fui perfeccionando para los diferentes contextos.” (P22)*

## DISCUSIÓN

Al estimular el pensamiento crítico sobre las competencias y habilidades en educación en salud, los estudiantes de pregrado pudieron presentar sus percepciones sobre el tema. En lo que respecta a las competencias, se observa la relación intrínseca con la obtención de conocimiento científico y con la capacidad de transformarlo en contenido accesible a los diferentes públicos, lo que exige no solo competencias, sino también habilidades educativas. Así, se puede afirmar que las competencias abarcan un conjunto de conocimientos, actitudes, motivaciones, autopercepciones y habilidades que hacen posible la realización de la acción educativa^(17)^.

A pesar de comprender el concepto de educación en salud, algunos estudiantes tuvieron cierta dificultad para correlacionar esta comprensión con las competencias necesarias para el profesional de enfermería en el ámbito de la promoción de la salud. Siguiendo una concepción alineada con la literatura nacional y las DCN de Enfermería, se entiende por competencia, en el ámbito de la formación, la movilización de conocimientos, habilidades, actitudes y valores, los cuales permiten al egresado adoptar una postura de liderazgo, comunicación, toma de decisiones y actuación como facilitador en acciones educativas^(3)^.

Aunque se reafirma la visión del enfermero como educador en salud, no siempre se evidencia la comprensión sobre las competencias que este profesional debe desarrollar para que dicho rol se consolide como herramienta de trabajo. Según los hallazgos de la literatura, las competencias deben estar relacionadas con el juicio clínico, la comunicación, la atención centrada en la persona, la equidad, la inclusión, la ética, la práctica basada en la evidencia, las políticas de salud y los determinantes sociales de la salud^(18)^.

En cuanto a las habilidades educativas, la comprensión se extendió a las capacidades prácticas, a la acción y al momento educativo propiamente dicho, describiéndose como la capacidad de transformar el conocimiento científico en conocimiento popular mediante diferentes metodologías. Al reflexionar sobre las habilidades requeridas del profesional de enfermería en el ejercicio de acciones educativas, se observa que estas se refieren a dimensiones prácticas, cognitivas y socioemocionales que orientan la movilización de las competencias en el trabajo en salud. Al fomentar el desarrollo de tales habilidades durante la carrera, se permite al futuro profesional un mejor desempeño asistencial y educativo^(19,20)^.

Además, se observó que la habilidad de comunicación fue la más citada entre los participantes de la investigación. Esta idea se ve reforzada por la literatura, en la que Daza-Betancourt et al.^(21)^ destacaron que la habilidad de comunicación asume un papel central, ya que, entre otras que debe desarrollar el enfermero, se vuelve esencial por permitir la creación de vínculos, confianza y empatía, además de facilitar la construcción de un ambiente propicio para el intercambio de información.

En relación con la categoría “Especificidades requeridas para el desempeño del profesional de enfermería como educador en salud con el público infantil”, se observó la preocupación de los estudiantes de pregrado por la promoción de la salud infantil a través de actividades educativas lúdicas, accesibles y adaptadas al público y a la realidad en la que se insertan los niños. Al pensar en la educación en salud, el entorno escolar se destaca por permitir la acción intersectorial entre salud y educación. Entre los profesionales de la salud, el enfermero asume el papel de protagonista como agente movilizador y articulador de las acciones educativas, ya que utiliza sus competencias para promover la prevención y la protección contra las enfermedades, con el objetivo de mitigar las vulnerabilidades y contribuir al desarrollo integral de los niños y adolescentes^(22)^.

Al considerar el contexto social, económico y de salud en el que se inserta el público, es posible planificar una acción que simule la vida cotidiana dentro de esa población, configurándose como un factor estimulante para que la niña o el niño se convierta en multiplicador de conocimientos en salud en la comunidad. Aunque lo lúdico actúa como facilitador del proceso de enseñanza-aprendizaje, es necesario ir más allá de un lenguaje accesible y alejado de los términos técnicos; se debe considerar el contexto de vida del niño, sus opiniones, intereses y razonamientos propios, para que sea posible sensibilizarlo, motivándolo a través de la relación de sus vivencias con el contenido abordado^(23)^.

Al planificar acciones de educación en salud, el facilitador, además del conocimiento científico sobre el tema a tratar, debe utilizar metodologías que constituyan puentes para la construcción del conocimiento junto al público-objetivo, así como prever y manejar posibles imprevistos durante la acción educativa.

En consonancia con los hallazgos de la investigación, se entiende que, para ejercer la actividad educativa en el ámbito infantil, es necesario planificar el momento educativo teniendo en cuenta el lugar, el público objetivo, la duración de la actividad y el contenido, con el fin de promover el conocimiento mediante el establecimiento de una relación de confianza^(24)^. Además, al definir que el público de la actividad educativa está compuesto por niños, el educador en salud debe considerar los diferentes enfoques correspondientes a las distintas etapas del desarrollo infantil, ya que el fracaso de algunas acciones educativas está relacionado con la generalización de materiales y métodos para diferentes grupos de edad, en desacuerdo con la literatura, que recomienda que la implementación de actividades educativas en la infancia debe considerar las etapas de desarrollo del niño^(25)^.

Para el desarrollo del proceso de enseñanza-aprendizaje, en el contexto infantil, los estudiantes de pregrado destacaron como esencial la articulación de la acción educativa con la vida cotidiana del niño, utilizando estrategias como teatro de marionetas, representaciones teatrales, narración de cuentos, parodias, videos educativos, dibujos, juguetes, musicoterapia, entre otras, ya que estas estrategias lúdicas garantizan la participación activa y facilitan el intercambio de conocimientos con el público objetivo. Desde esta perspectiva, la literatura señala que estas estrategias presentan potencial de aprendizaje, principalmente porque el juego permite la simulación de escenarios y contextos, donde los niños pueden explorar, resolver dudas y construir un repertorio sobre el tema abordado^(26)^.

Entre las estrategias educativas mencionadas, además de las que simulan la vida cotidiana de la niña o el niño, se destacaron estrategias que estimulan el protagonismo infantil, como la creación de círculos de conversación, en los que se anima a las niñas y los niños a hablar, expresar opiniones y presentar ejemplos.

Al utilizar estrategias que promueven el protagonismo infantil, se pone de manifiesto el éxito de la actividad educativa, ya que, aunque el uso de materiales lúdicos está ampliamente difundido, el éxito de la intervención en salud reside en las relaciones establecidas durante el proceso, sin limitarse al uso de objetos en el momento educativo^(23)^.

Además, entre los recursos utilizados para garantizar la participación activa del público infantil, los estudiantes destacaron el uso de juegos educativos, tanto tradicionales como digitales, con énfasis en el desarrollo de tecnologías educativas virtuales. Se observó una preferencia por el desarrollo y la aplicación de juegos educativos digitales con niños, ya que estos estimulan el aprendizaje mediante la valorización de las experiencias previas, favorecen el diálogo entre los facilitadores y el público objetivo y contribuyen al desarrollo de aspectos emocionales, cognitivos y motores, además de distanciarse de las metodologías tradicionales de enseñanza^(27)^.

También se evidenció, en los resultados de la investigación, el impacto de la pandemia de COVID-19 sobre las acciones de promoción de la salud dirigidas al público infantil, lo que hizo necesaria la reformulación de las metodologías tradicionalmente utilizadas para viabilizar la continuidad de las acciones educativas. En este contexto, se introdujo el uso de las Tecnologías de la Información y la Comunicación (TIC), que comprenden recursos electrónicos que permiten comunicarse, interactuar y aprender, tales como *software* educativo, videos educativos, juegos, *sitios web* e imágenes, lo que permite promover la salud más allá de las barreras físicas^(28)^.

En los resultados de la investigación, se evidenció una limitación en la expresión de la comprensión sobre competencias y habilidades, así como también sobre las estrategias y los recursos a utilizar en actividades educativas con el público infantil, entre los estudiantes de pregrado que no presentaban experiencia en extensión o actuación práctica. La inserción en el entorno escolar, la escucha de las demandas de los alumnos y la integración con los docentes y gestores contribuyen a una mayor seguridad y al desarrollo de *expertise* en la realización de acciones educativas, lo que resalta la importancia del trípode universitario.

Se destacó la integración entre la enseñanza, la investigación y la extensión promovida en el entorno académico como la principal contribución al desarrollo de competencias y habilidades orientadas a las acciones de educación en salud, ya que permite la inserción de los estudiantes de pregrado en entornos teórico-prácticos, favoreciendo una visión multidimensional, el desarrollo de una mirada clínica crítica y la contextualización del conocimiento para la transformación social^(29)^. Además, los estudiantes de pregrado mencionaron la importancia de la tutoría académica como estrategia para mejorar los conocimientos adquiridos en la asignatura y desarrollar habilidades de enseñanza-aprendizaje, ya que la tutoría tiene como propósito aclarar dudas relacionadas con el área de conocimiento, ayudar en la profundización teórica y reforzar las habilidades prácticas aprendidas durante la asignatura^(30)^.

Cabe destacar la insatisfacción de los estudiantes de pregrado con respecto al plan de estudios, quienes resaltan una mayor proximidad de las disciplinas con enfermedades y afecciones y un alejamiento de la perspectiva de la educación popular en salud, especialmente en relación con el público infantil.

En los discursos se evidenció la necesidad de ajustes curriculares, considerando que, para capacitar mejor a los futuros enfermeros, el plan de estudios de la licenciatura debe estar alineado con las transformaciones en la prestación de cuidados de salud para los diferentes públicos. En este sentido, el ambiente académico se configura como el espacio de transformación y alineación con las necesidades actuales y futuras en el ámbito de la salud^(17)^.

Aunque el desarrollo de las competencias y habilidades del enfermero se produce de manera gradual, a partir de la acumulación de experiencias con diferentes públicos y de la reinterpretación de los conocimientos ante nuevas situaciones, los estudiantes informaron de dificultades e inseguridad a la hora de promover acciones educativas dirigidas al público infantil, debido al mayor enfoque de la carrera en el público adulto y de la tercera edad. En este sentido, para reducir dicha inseguridad, se debe estimular a los estudiantes de pregrado durante la formación a desarrollar autonomía en la adquisición de conocimientos en salud, a mejorar la comunicación, a fortalecer las relaciones interpersonales y a la capacidad de resolución^(31)^.

Para que esto ocurra, las instituciones de educación superior pueden adoptar metodologías activas de aprendizaje en la formación profesional, ya que estas simulan o insertan al estudiante en el campo de actuación con el objetivo de facilitar la adquisición de habilidades en salud^(32)^. Entre estas estrategias, destaca el uso de tecnologías educativas digitales, que favorecen el desarrollo cognitivo y actitudinal necesario en el contexto de la promoción de la salud, además de diversificar el proceso de enseñanza-aprendizaje^(30)^.

Aunque este estudio ha contribuido a la comprensión de las competencias y habilidades necesarias para que los estudiantes de Enfermería actúen como educadores en salud en la atención de la salud infantil, se observó que la inclusión únicamente de universidades públicas y de un solo estado constituye una limitación para la profundización del tema. Además, se identificaron las lagunas encontradas en la literatura sobre la conceptualización de las competencias en educación en salud y de las habilidades educativas en el contexto de la enfermería, lo que restringió una discusión más específica y comparativa de los hallazgos. Por lo tanto, se sugiere que los estudios futuros amplíen la comprensión de estos conceptos, abarcando un mayor número de instituciones de educación superior, incluidas las privadas, que concentran una parte significativa de los estudiantes de grado de Enfermería.

## CONCLUSIÓN

La percepción de los estudiantes de enfermería sobre las competencias y habilidades necesarias para la educación en salud en el contexto infantil pone de manifiesto la existencia de un déficit en la formación curricular, especialmente en lo que se refiere a la preparación para actuar como educadores en salud de niños. Aunque demuestran comprender la importancia de articular el contenido científico con metodologías lúdicas para transformarlo en conocimiento accesible y significativo, los estudiantes revelaron inseguridad y dificultad para aplicar estas prácticas, lo que se atribuye a la escasa experiencia con el público infantil durante la carrera.

Sin embargo, para los estudiantes de pregrado con experiencia teórico-práctica en el contexto escolar, la competencia puede definirse como la articulación integrada entre conocimientos, habilidades y actitudes, requerida para que el profesional movilice lo que sabe (conocimiento) y sepa cómo hacerlo (habilidad), de manera contextualizada y adecuada al público y a la situación vivida. Cabe destacar, además, la importancia de la sensibilidad del estudiante para reconocer y valorar las especificidades de cada público, teniendo en cuenta sus necesidades reales y el compromiso de propiciar un espacio lúdico y creativo, capaz de motivar y estimular la participación de los niños.

En este escenario, se destacan las actividades de extensión, los grupos de investigación y la tutoría académica como espacios privilegiados para el desarrollo de estas competencias, ya que promueven la inserción de los estudiantes en contextos reales y estimulan la integración entre la teoría y la práctica. Así, tales experiencias complementan las lagunas curriculares y contribuyen a la formación de enfermeros más preparados para actuar como educadores en salud en el cuidado infantil.

## Data Availability

El conjunto de datos que respaldan las conclusiones de este estudio está disponible previa solicitud al autor correspondiente.
